# A novel and efficient synthesis of phenanthrene derivatives via palladium/norbornadiene-catalyzed domino one-pot reaction

**DOI:** 10.3762/bjoc.15.26

**Published:** 2019-01-31

**Authors:** Yue Zhong, Wen-Yu Wu, Shao-Peng Yu, Tian-Yuan Fan, Hai-Tao Yu, Nian-Guang Li, Zhi-Hao Shi, Yu-Ping Tang, Jin-Ao Duan

**Affiliations:** 1 National and Local Collaborative Engineering Center of Chinese Medicinal Resources Industrialization and Formulae Innovative Medicine, Jiangsu Collaborative Innovation Center of Chinese Medicinal Resources Industrialization, Jiangsu Key Laboratory for High Technology Research of TCM Formulae, Nanjing University of Chinese Medicine, Nanjing 210023, China; 2 Department of Nuclear Medicine, Nanjing First Hospital, Nanjing Medical University, Nanjing 21006, China; 3 Department of Organic Chemistry, China Pharmaceutical University, Nanjing 211198, China; 4 College of Pharmacy and Shaanxi Collaborative Innovation Center of Chinese Medicinal Resources Industrialization, Shaanxi University of Chinese Medicine, Xi’an 712046, Shaanxi Province, China

**Keywords:** C–H activation, norbornadiene, palladium, phenanthrene derivatives

## Abstract

Herein we report a novel palladium-catalyzed reaction that results in phenanthrene derivatives using aryl iodides, *ortho*-bromobenzoyl chlorides and norbornadiene in one pot. This dramatic transformation undergoes *ortho-*C–H activation, decarbonylation and subsequent a retro-Diels–Alder process. Pleasantly, this protocol has a wider substrate range, shorter reaction times and higher yields of products than previously reported methods.

## Introduction

**Figure 1:**
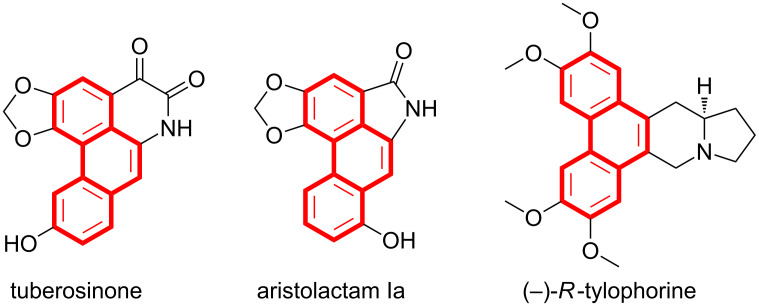
Representative natural products containing a phenanthrene moiety.

Phenanthrene is a polycyclic aromatic hydrocarbon which contains three benzene rings. The phenanthrenes can be used as fundamental building blocks or intermediates in the synthesis of complex natural products such as tuberosinone [1], aristolactam Ia [2] and (−)-*R*-tylophorine [3] (Figure 1). Meanwhile, they also demonstrate a wide range of biological activities including anticancer [4], anti-HIV [5], antibacterial [6], anti-inflammatory [7] and so on.

**Scheme 1:**
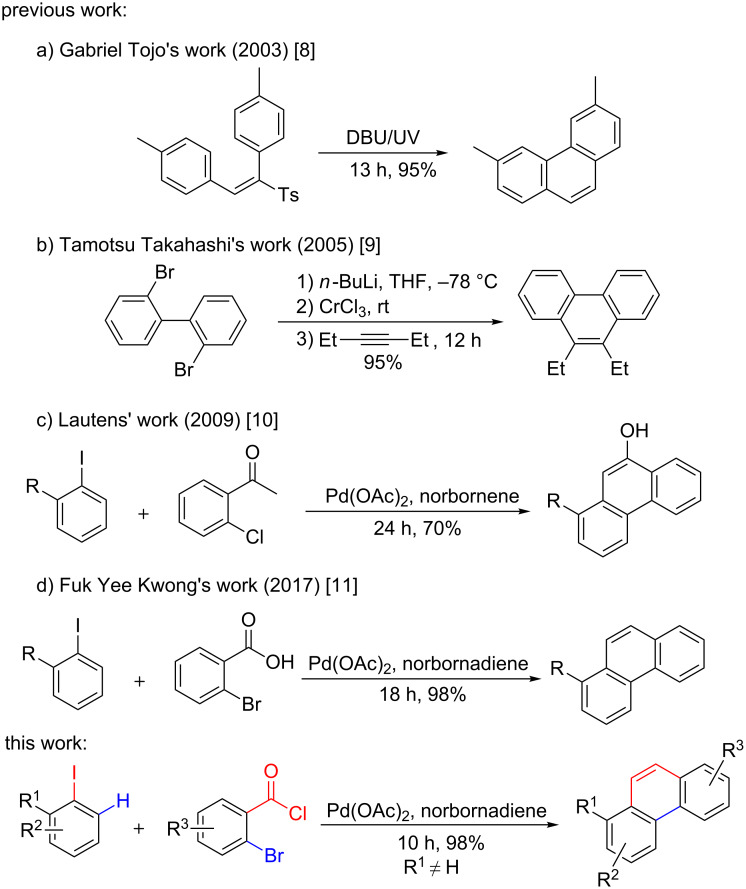
Different methods for the synthesis of phenanthrene derivatives.

During the past decades, numerous methods for the preparation of phenanthrene derivatives have been developed. In 2003, Gabriel Tojo’s group reported a base-catalyzed photochemical synthesis of phenanthrene derivatives [8] through intramolecular aromatic coupling (Scheme 1a). Although this method offered an atom-economic and easier strategy for the construction of phenanthrene derivatives, the starting materials in this reaction were difficult to obtain, which limited the development of this approach. Moreover, the ultraviolet light (UV) used in this reaction may cause skin damage to experimenters. Alternatively, alkynes (Scheme 1b) played an important role in the synthesis of phenanthrene scaffolds under transition metal catalysis [9]. However, this protocol required complicated procedures, harsh reaction conditions and was incompatible with many functional groups. Subsequently, facile one-pot approaches had been realized via norbornene-mediated palladium-catalyzed Catellani reaction by Lautens’ group [10] (Scheme 1c). Though this strategy was superior to previous methods in terms of mildness of the reaction conditions, it had a limited substrate scope and relatively low reaction efficiency. Very recently, Fuk Yee Kwong and co-workers (Scheme 1d) developed a straightforward one pot π-extension method using norbornadiene instead of norbornene as directing group to afford the phenanthrenes [11]. However, *ortho*-haloaryl carboxylic acids employed in this approach had low reactivity, which needed higher reaction temperatures and longer reaction time. Therefore, the development of novel, efficient, and highly functional group tolerant methods for the synthesis of phenanthrene derivatives is still desirable.

Domino reactions such as norbornene-mediated palladium-catalyzed Catellani reactions, which were originally discovered by Catellani in the 1990s [12] and further developed by groups of Catellani and Lautens et al. [13–17], hold great potential for not only settling the sequential reactions in one pot, but also providing access to multisubstituted arenes. In the previous work, we innovatively developed a strategy for the remote C–H alkylation of arenes [18]. Recently, our group also achieved aromatic ketones [19] and 2-alkynyl aromatic ketones [20] successfully through *ortho*-acylation and *ipso*-Suzuki coupling or alkynylation for the aryl iodides. In this paper, we developed an efficient domino reaction of aryl iodides with *ortho*-bromobenzoyl chlorides and norbornadiene leading to phenanthrene derivatives, which could be widely used in the synthesis of vital intermediates for functional materials, pharmaceutical agents and natural products.

## Results and Discussion

We initiated our investigations by evaluating the three-component cross-coupling reaction of 2-iodotoluene (**1a**), *ortho*-bromobenzoyl chloride (**2a**) and norbornadiene, and we optimized the reaction conditions [21]. Firstly, the reaction took place under Pd(PPh_3_)_4_/PPh_3_ as the catalyst, Cs_2_CO_3_ as base, and dimethyl formamide (DMF) as solvent at 105 °C for 10 h under N_2_ atmosphere. Meanwhile, different pallladium species were tested in this reaction system. It was found that Pd(OAc)_2_ was the most effective palladium catalyst, and the desired product **y-1** was obtained in 98% yield (Table 1, entries 1–4). Next, we studied the influence of different ligands in terms of electron-rich, electron-deficient substituents and steric hindrance. The results suggested that different ligands had little effect on this reaction (Table 1, entries 5–7). After a further opimization of various bases, we found that K_2_CO_3_ owned a slimilar reactivity as Cs_2_CO_3_, while CsOAc with weaker alkalinity showed a lower activity than K_2_CO_3_, giving a yield of 62% (Table 1, entries 8 and 9). Besides, organic bases such as Et_3_N and *N*,*N*-diisopropylethylamine (DIPEA) were not effective for this reaction (Table 1, entries 10 and 11). Furthermore, different solvents were then tested, including MeCN, toluene, 1,4-dioxane, tetrahydrofuran (THF), and dimethylacetamide (DMAC), and we found the yield of **y-1** was inferior to that done by DMF (Table 1, entries 12–16).

**Table 1:** Optimization of the reaction conditions.^a^

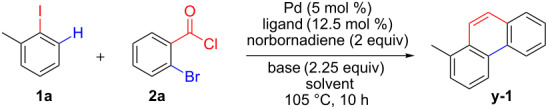					

entry	catalyst	ligand	base	solvent	yield [%]^b^

1	Pd(PPh_3_)_4_	PPh_3_^c^	Cs_2_CO_3_	DMF	86
2	PdCl_2_	PPh_3_	Cs_2_CO_3_	DMF	75
3	Pd(OAc)_2_	PPh_3_	Cs_2_CO_3_	DMF	98
4	Pd(PPh_3_)_2_Cl_2_	PPh_3_	Cs_2_CO_3_	DMF	82
5	Pd(OAc)_2_	TFP^d^	Cs_2_CO_3_	DMF	95
6	Pd(OAc)_2_	diethyl maleate	Cs_2_CO_3_	DMF	94
7	Pd(OAc)_2_	X-PHOS^e^	Cs_2_CO_3_	DMF	96
8	Pd(OAc)_2_	PPh_3_	K_2_CO_3_	DMF	95
9	Pd(OAc)_2_	PPh_3_	CsOAc	DMF	62
10	Pd(OAc)_2_	PPh_3_	Et_3_N	DMF	trace
11	Pd(OAc)_2_	PPh_3_	DIPEA	DMF	trace
12	Pd(OAc)_2_	PPh_3_	Cs_2_CO_3_	MeCN	N.D.
13	Pd(OAc)_2_	PPh_3_	Cs_2_CO_3_	toluene	64
14	Pd(OAc)_2_	PPh_3_	Cs_2_CO_3_	1,4-dioxane	68
15	Pd(OAc)_2_	PPh_3_	Cs_2_CO_3_	THF	trace
16	Pd(OAc)_2_	PPh_3_	Cs_2_CO_3_	DMAC	92

^a^Reaction conditions: **1a** (0.3 mmol, 1.0 equiv), **2a** (0.36 mmol, 1.2 equiv), norbornadiene (0.6 mmol, 2.0 equiv), Pd (5 mol %), ligand (12.5 mol %), base (0.675 mmol, 2.25 equiv), solvent (4 mL), 105 °C for 10 h under N_2_. ^b^Yield of isolated product. N.D. = not determined. ^c^PPh_3_ = triphenylphosphine. ^d^TFP = tris(2-furyl)phosphine. ^e^X-PHOS = 2-dicyclohexylphosphino-2’,4’,6’-triisopropylbiphenyl.

**Scheme 2:**
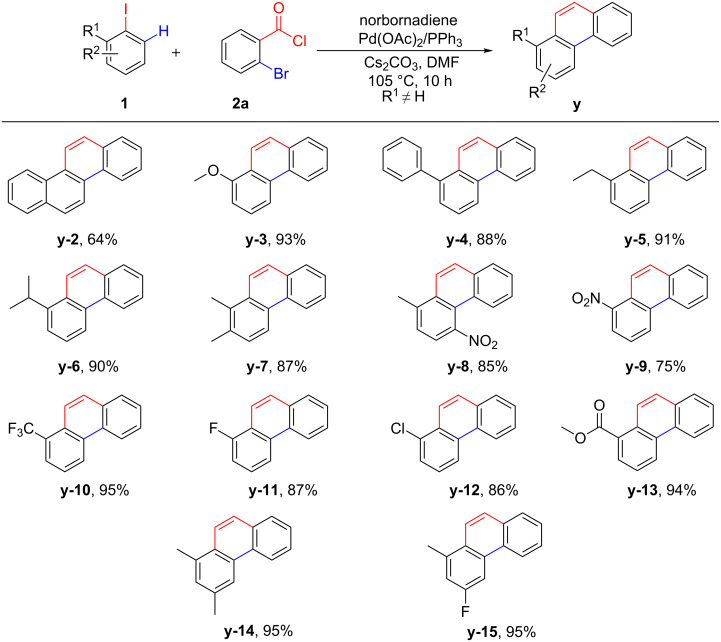
Substrate scope with various aryl iodides. Reaction conditions: **1** (0.3 mmol, 1.0 equiv), **2a** (0.36 mmol, 1.2 equiv), norbornadiene (0.6 mmol, 2.0 equiv), Pd(OAC)_2_ (5 mol %), PPh_3_ (12.5 mol %), Cs_2_CO_3_ (0.675 mmol, 2.25 equiv), DMF (4 mL), 105 °C for 10 h under N_2_.

With the optimum reaction conditions in hand (Table 1, entry 3), we expanded the aryl iodide substrates of this reaction (Scheme 2). As a result, it was found that both electron-deficient and electron-rich aryl iodides progressed well in the transformation, and the yield of relevant phenanthrene derivatives **y-2**–**y-15** was quite well. Therefore, it is speculated that the intrinsic electronic properties of aryl iodides makes no difference in this process. The reaction of methoxy-, 2-ethyl-, 2-isopropyl, and 2-phenyliodobenzenes in this procedure afforded the desired products in excellent yields (**y-3**, **y-5**, **y-6** and **y-4**), while 1-iodonaphthalene gave a relative low yield of 64% (**y-2**). Gratifyingly, the reaction of disubstituted iodine substrates proceeded smoothly to deliver targeted compounds **y-7**, **y-8**, **y-14** and **y-15** as well. Introducing electron-withdrawing groups at the *ortho-*position of aryl iodides, such as nitro, fluorine, chlorine, trifluoromethyl and ester, gave 75–95% yields of the corresponding products **y-9**–**y-13**, which were precursors for further transition metal-catalyzed cross-coupling reactions.

**Scheme 3:**
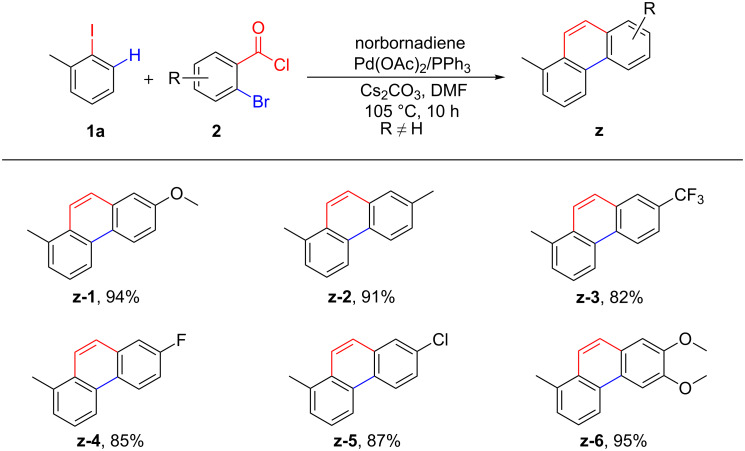
Scope of the reaction in terms of *ortho*-bromobenzoyl chlorides. Reaction conditions: **1a** (0.3 mmol, 1.0 equiv), **2** (0.36 mmol, 1.2 equiv), norbornadiene (0.6 mmol, 2.0 equiv), Pd(OAC)_2_ (5 mol %), PPh_3_ (12.5 mol %), Cs_2_CO_3_ (0.675 mmol, 2.25 equiv), DMF (4 mL), 105 °C for 10 h under N_2_.

To further explore the generality of this reaction, different substituted *ortho*-bromobenzoyl chlorides were then tested (Scheme 3). The *ortho*-bromobenzoyl chlorides possessing electron-donating groups, such as methyl and methoxy, were well tolerated in this transformation, and the targeted components were acquired in excellent yields (**z-1** and **z-2**). Notably, *ortho*-bromobenzoyl chlorides with electron-withdrawing substituents were also compatible substrates, which afforded the corresponding products in 82–87% yield (**z-3**–**z-5**). At the same time, a disubstituted substrate was tolerated well to this conversion (**z-6**). However, the substrates containing *ortho-*substituents were disadvantageous for this reaction, and none of the desired products was obtained, which indicated that the reaction was strongly influenced by steric hindrance.

**Scheme 4:**
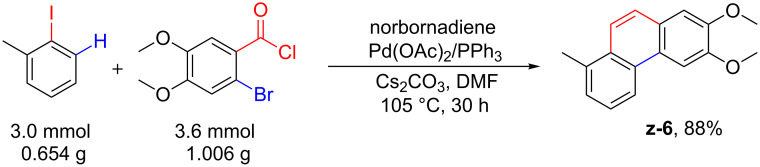
Gram scale synthesis of **z-6**.

It was noteworthy that this sequential one-pot reaction could be carried out on the gram scale (Scheme 4). We selected 2-iodotoluene and 2-bromo-4,5-dimethoxybenzoyl chloride as substrates. When the reaction of 2-iodotoluene (**1a**, 3.0 mmol, 1.0 equiv, 0.654 g), 2-bromo-4,5-dimethoxybenzoyl chloride (3.6 mmol, 1.2 equiv, 1.006 g), and norbornadiene (6.0 mmol, 2.0 equiv, 0.553 g) was performed in the presence of 0.15 mmol of Pd(OAc)_2_, 0.375 mmol of PPh_3_ and 6.75 mmol of Cs_2_CO_3_ at 105 °C in DMF under N_2_ for 30 h, the desired compound **z-6** was isolated in 88% yield.

**Scheme 5:**
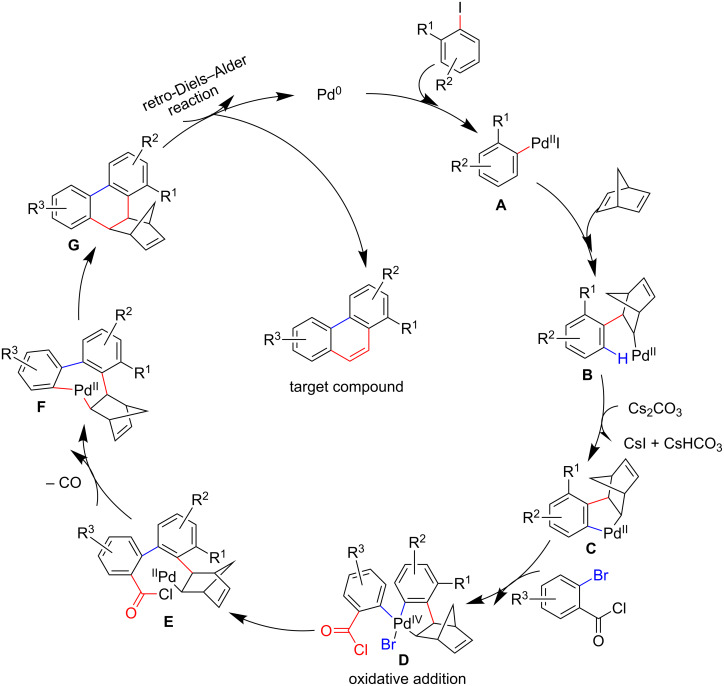
Proposed mechanism for the formation of phenanthrene derivatives.

Based on the above experimental results and the use of norbornadiene in Catellani reactions followed by retro-Diels–Alder reaction firstly reported by Lautens et al. [22–24], which is mentioned in recent works [11], a proposed mechanism for this domino reaction is presented in Scheme 5. As is commonly considered, the aryl-Pd^II^ complex **A** is formed by oxidative addition of aryl iodide to the Pd^0^ complex, which is followed by the insertion of norbornadiene to the C–Pd bond of **A** to produce **B**. Then, an *ortho-*C–H activation reaction occurs to **B**, which offers compound **C** with a five-membered palladacycle. **C** undergoes the process of oxidative addition with *ortho*-bromobenzoyl chloride to give the Pd^IV^ intermediate **D**, and **E** can be obtained via a reductive elimination reaction. A novel aryl-Pd^II^ species **F** is formed through removing carbon monoxide from **E**. Ultimately, **G** will experience immediate retro-Diels–Alder reaction after the catalytic cycle to afford the target product while taking off cyclopentadiene.

## Conclusion

In summary, we have developed a novel and efficient protocol which allows us to construct a variety of phenanthrene derivatives starting from aryl iodides, *ortho*-bromobenzoyl chlorides and norbornadiene in one pot. A wide range of functional groups are compatible with the reaction, including both electron-withdrawing and electron-donating groups. Compared with previous work for the synthesis of the phenanthrenes, this method shows higher reactivity, shorter reaction times, and higher yields of the target compounds. Meanwhile, these flexible approaches to the phenanthrene derivatives would be expected to provide significant references to material chemistry, pharmaceutical agents and natural product synthesis.

## Experimental

### General remarks

All reactions were carried out under a nitrogen atmosphere unless otherwise stated and commercially available reagents were used without further purification. Solvents were purified by standard techniques without special instructions. Thin-layer chromatography (TLC) was performed on GF254 plates, and the spots were monitored through UV light. Flash chromatography was carried out on silica gel 300–400 mesh. ^1^H and ^13^C NMR spectra were recorded in CDCl_3_ at 500 and 126 MHz, respectively, using the solvents as internal standards. High-resolution mass spectra were taken on Waters Synapt MS. The peak patterns are indicated as follows: s, singlet; d, doublet; t, triplet; m, multiplet; q, quartet. The coupling constants (*J*) are offered in Hz. The *ortho*-bromobenzoyl chlorides **2a**–**f** were synthesized using the known method [25–26].

### General procedure for the preparation of products

A dried round-bottomed flask was charged with aryl iodide (0.30 mmol, 1.0 equiv), *ortho*-bromobenzoyl chlorides (0.36 mmol, 1.2 equiv), norbornadiene (0.60 mmol, 2.0 equiv), Pd(OAc)_2_ (5 mol %), triphenylphosphine (12.5 mol %), Cs_2_CO_3_ (0.675 mmol, 2.25 equiv), and DMF (4 mL). The mixture was stirred at 105 °C under nitrogen atmosphere for 10 h. After cooling to room temperature, the mixture was diluted with ethyl acetate (5 mL) and brine (10 mL), and extracted with ethyl acetate (3 × 10 mL). The combined organic phase was washed with brine, dried with anhydrous Na_2_SO_4_, filtered, and concentrated under reduced pressure. The crude product was purified by column chromatography on silica gel (petroleum ether/ethyl acetate as eluent) to afford the target compounds.

## Supporting Information

File 1 Spectral data of products, and ^1^H NMR and ^13^C NMR spectra for the products.
