# Supplementary material for: A novel and efficient synthesis of phenanthrene derivatives via palladium/norbornadiene-catalyzed domino one-pot reaction
Source: Beilstein J Org Chem. 2019 Jan 31;15:291–8. doi: 10.3762/bjoc.15.26 (PMC6369988; doi:10.3762/bjoc.15.26)
Supplement: File 1 — Spectral data of products, and 1H NMR and 13C NMR spectra for the products. [file Beilstein_J_Org_Chem-15-291-s001.pdf]

BEILSTEIN JOURNAL OF ORGANIC CHEMISTRY

Supporting Information

for

A novel and efficient synthesis of phenanthrene derivatives via palladium/norbornadiene-catalyzed domino one-pot reaction

Yue Zhong, Wen-Yu Wu, Shao-Peng Yu, Tian-Yuan Fan, Hai-Tao Yu, Nian-Guang Li, Zhi-Hao Shi, Yu-Ping Tang and Jin-Ao Duan

Beilstein J. Org. Chem. **2019,** *15,* 291–298. doi:10.3762/bjoc.15.26

Spectral data of products, and ^{1}H NMR and ^{13}C NMR spectra for the products

License and Terms: This is a supporting information file under the terms of the Creative Commons Attribution License (http://creativecommons.org/licenses/by/4.0). Please note that the reuse, redistribution and reproduction in particular requires that the authors and source are credited.
The license is subject to the *Beilstein Journal of Organic Chemistry* terms and conditions: (https://www.beilstein-journals.org/bjoc)

Table of contents

1. Spectral data of products...S2

2. ^{1}H NMR and ^{13}C NMR spectra for the products...............................S8

1. Spectral data of products

1-Methylphenanthrene (y-1)

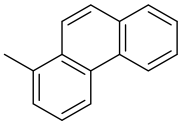

Following the general procedure on a 0.30 mmol scale, compound **y-1** (56.5 mg, 98 %) was obtained as white solid. **^{1}H NMR** (500 MHz, $CDCl_3$) δ 8.71 (d, *J* = 8.2 Hz, 1H), 8.59 (d, *J* = 8.3 Hz, 1H), 7.96 (d, *J* = 9.1 Hz, 1H), 7.90 (d, *J* = 8.9 Hz, 1H), 7.79 (d, *J* = 9.1 Hz, 1H), 7.68 – 7.62 (m, 1H), 7.61 – 7.57 (m, 1H), 7.57 – 7.52 (m, 1H), 7.45 (d, *J* = 7.1 Hz, 1H), 2.76 (s, 3H). **^{13}C NMR** (126 MHz, $CDCl_3$) δ 134.91, 131.70, 130.85, 130.71, 130.39, 128.51, 127.80, 126.72, 126.58, 126.45, 126.18, 122.99, 122.90, 120.90, 19.99. HRMS (ESI) calcd. for $C_{15}H_{12}$ $[M+H]^+$:193.1017, found 193.1014; $[M+Na]^+$: 215.0837, found 215.0835.

Chrysene (y-2)

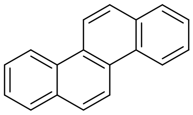

Following the general procedure on a 0.30 mmol scale, compound **y-2** (43.8 mg, 64 %) was obtained as white solid. **^{1}H NMR** (500 MHz, $CDCl_3$) δ 8.82 (d, *J* = 8.4 Hz, 2H), 8.76 (d, *J* = 9.0 Hz, 2H), 8.04 (t, *J* = 8.2 Hz, 4H), 7.74 (m, *J* = 8.3, 7.0, 1.3 Hz, 2H), 7.71 – 7.64 (m, 2H). **^{13}C NMR** (126 MHz, $CDCl_3$) δ 132.18, 130.56, 128.57, 128.22, 127.35, 126.68, 126.38, 123.16, 121.22. HRMS (ESI) calcd. for $C_{18}H_{12}$ $[M+H]^+$:229.1017, found 229.1012; $[M+Na]^+$: 251.0837, found 251.0834.

1-Methoxyphenanthrene (y-3)

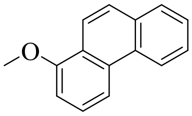

Following the general procedure on a 0.30 mmol scale, compound **y-3** (58.1 mg, 93 %) was obtained as white solid. **^{1}H NMR** (500 MHz, $CDCl_3$) δ 8.70 (d, *J* = 8.2 Hz, 1H), 8.31 (d, *J* = 8.4 Hz, 1H), 8.26 (d, *J* = 9.1 Hz, 1H), 7.92 (dd, *J* = 7.9, 1.1 Hz, 1H), 7.78 (d, *J* = 9.1 Hz, 1H), 7.68 – 7.58 (m, 3H), 7.04 (d, *J* = 7.8 Hz, 1H), 4.07 (s, 3H). **^{13}C NMR** (126 MHz, $CDCl_3$) δ 154.99, 131.27, 130.54, 129.09, 127.56, 125.69, 125.62, 125.43, 125.08, 122.22, 119.39, 114.04, 104.86, 54.76. HRMS (ESI) calcd. for $C_{15}H_{12}O$ $[M+H]^+$:209.0966, found 209.0970; $[M+Na]^+$: 231.0786, found 231.0789.

1-Phenylphenanthrene (y-4)

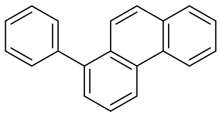

Following the general procedure on a 0.30 mmol scale, compound **y-4** (67.14 mg, 88 %) was obtained as colorless oil. ^{1}**H NMR** (500 MHz, $CDCl_3$) δ 8.78 (dd, *J* = 8.3, 4.6 Hz, 2H), 7.91 (d, *J* = 7.8 Hz, 1H), 7.84 (d, *J* = 9.2 Hz, 1H), 7.75 – 7.69 (m, 3H), 7.66 – 7.62 (m, 1H), 7.58 (dd, *J* = 7.2, 1.0 Hz, 1H), 7.54 (d, *J* = 4.3 Hz, 4H), 7.51 – 7.46 (m, 1H). ^{13}**C NMR** (126 MHz, $CDCl_3$) δ 141.14, 141.04, 131.76, 130.71, 130.44, 130.25, 129.95, 128.49, 128.30, 127.92, 127.27, 126.89, 126.72, 126.68, 125.98, 124.63, 122.99, 122.17. HRMS (ESI) calcd. for $C_{20}H_{14}$ $[M+H]^+$:255.1174, found 255.1178; $[M+Na]^+$: 277.0993, found 277.0995.

1-Ethylphenanthrene (y-5)

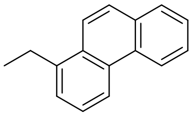

Following the general procedure on a 0.30 mmol scale, compound **y-5** (56.3 mg, 91 %) was obtained as white solid. ^{1}**H NMR** (500 MHz, $CDCl_3$) δ 8.74 (d, *J* = 7.8 Hz, 1H), 8.63 (d, *J* = 8.4 Hz, 1H), 8.05 (d, *J* = 9.2 Hz, 1H), 7.93 (dd, *J* = 7.8, 1.3 Hz, 1H), 7.81 (d, *J* = 9.2 Hz, 1H), 7.75 – 7.56 (m, 3H), 7.51 (d, *J* = 7.1 Hz, 1H), 3.21 (q, *J* = 7.5 Hz, 2H), 1.45 (t, *J* = 7.6 Hz, 3H). ^{13}**C NMR** (126 MHz, $CDCl_3$) δ 141.05, 131.67, 130.91, 130.68, 130.08, 128.46, 126.73, 126.56, 126.46, 126.36, 126.22, 123.01, 122.58, 120.91, 15.54. HRMS (ESI) calcd. for $C_{16}H_{14}$ $[M+H]^+$:207.1174, found 207.1179; $[M+Na]^+$: 229.0993, found 277.0999.

1-Isopropylphenanthrene (y-6)

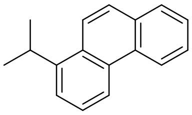

Following the general procedure on a 0.30 mmol scale, compound **y-6** (59.5 mg, 90 %) was obtained as white solid. ^{1}**H NMR** (500 MHz, $CDCl_3$) δ 8.76 (d, *J* = 8.2 Hz, 1H), 8.65 (d, *J* = 8.2 Hz, 1H), 8.14 (d, *J* = 9.3 Hz, 1H), 7.94 (d, *J* = 7.7 Hz, 1H), 7.83 (d, *J* = 9.3 Hz, 1H), 7.71 – 7.60 (m, 4H), 3.88 (m, 1H), 1.49 (d, *J* = 6.8 Hz, 6H). ^{13}**C NMR** (126 MHz, $CDCl_3$) δ 145.27, 131.49, 130.94, 130.64, 129.58, 128.33, 126.64, 126.51, 126.42, 126.34, 123.01, 122.73, 122.08, 120.65, 28.84, 23.75. HRMS (ESI) calcd. for $C_{17}H_{16}$ $[M+H]^+$:221.1330, found 221.1327; $[M+Na]^+$: 243.1150, found 243.1148.

1,2-Dimethylphenanthrene (y-7)

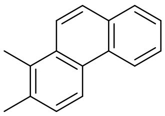

Following the general procedure on a 0.30 mmol scale, compound **y-7** (53.8 mg, 87 %) was obtained as white solid. ^{1}**H NMR** (500 MHz, $CDCl_3$) δ 8.71 (d, *J* = 8.3 Hz, 1H), 8.52 (d, *J* = 8.5 Hz, 1H), 8.06 (d, *J* = 9.2 Hz, 1H), 7.92 (d, *J* = 7.9 Hz, 1H), 7.80 (d, *J* = 9.2 Hz, 1H), 7.67 (m, 1H), 7.62 – 7.58 (m, 1H), 7.51 (d, *J* = 8.5 Hz, 1H), 2.70 (s, 3H), 2.57 (s, 3H). ^{13}**C NMR** (126 MHz, $CDCl_3$) δ 134.39, 132.31, 131.14, 130.94, 130.78, 129.08, 128.73, 128.31, 126.64, 126.44, 126.06, 122.88, 122.69, 120.19, 20.91, 15.04. HRMS (ESI) calcd. for $C_{16}H_{14}$ $[M+H]^+$:207.1174, found 207.1171; $[M+Na]^+$: 229.0993, found 229.0989.

1-Methyl-4-nitrophenanthrene (y-8)

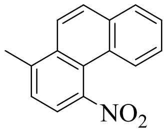

Following the general procedure on a 0.30 mmol scale, compound **y-8** (60.5 mg, 85 %) was obtained as light yellow solid. **^{1}H NMR** (500 MHz, $CDCl_3$) δ 9.48 (s, 1H), 8.76 (d, *J* = 8.3 Hz, 1H), 8.25 (s, 1H), 8.00 – 7.96 (m, 3H), 7.81 – 7.76 (m, 1H), 7.74 – 7.70 (m, 1H), 2.85 (s, 3H). **^{13}C NMR** (126 MHz, $CDCl_3$) δ 145.33, 137.02, 134.61, 131.90, 130.85, 130.80, 130.00, 128.89, 127.87, 127.81, 123.15, 122.05, 121.00, 117.23, 20.17. HRMS (ESI) calcd. for $C_{15}H_{11}NO_2$ $[M+H]^+$:238.0868, found 238.0872; $[M+Na]^+$: 260.0687, found 260.0683.

1-Nitrophenanthrene (y-9)

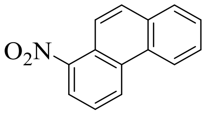

Following the general procedure on a 0.30 mmol scale, compound **y-9** (50.2 mg, 75 %) was obtained as light yellow solid. **^{1}H NMR** (500 MHz, $CDCl_3$) δ 8.99 (d, *J* = 8.4 Hz, 1H), 8.71 (d, *J* = 8.2 Hz, 1H), 8.32 (d, *J* = 9.3 Hz, 1H), 8.21 (d, *J* = 8.7 Hz, 1H), 7.98 (d, *J* = 9.1 Hz, 2H), 7.79 – 7.70 (m, 3H). **^{13}C NMR** (126 MHz, $CDCl_3$) δ 147.87, 131.77, 131.59, 130.63, 129.24, 128.82, 127.96, 127.93, 127.78, 125.12, 124.18, 123.24, 122.92, 120.12. HRMS (ESI) calcd. for $C_{14}H_9NO_2$ $[M+H]^+$:224.0712, found 224.0716; $[M+Na]^+$: 246.0531, found 246.0537.

1-(Trifluoromethyl)phenanthrene (y-10)

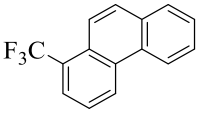

Following the general procedure on a 0.30 mmol scale, compound **y-10** (70.2 mg, 95 %) was obtained as white solid. **^{1}H NMR** (500 MHz, $CDCl_3$) δ 8.92 (d, *J* = 8.4 Hz, 1H), 8.71 (d, *J* = 8.2 Hz, 1H), 8.14 (dd, *J* = 9.3, 1.9 Hz, 1H), 8.00 (d, *J* = 7.4 Hz, 1H), 7.96 (d, *J* = 9.0 Hz, 1H), 7.91 (d, *J* = 9.3 Hz, 1H), 7.76 – 7.67 (m, 3H). **^{13}C NMR** (126 MHz, $CDCl_3$) δ 130.49, 130.21, 128.88, 127.84, 127.61, 127.29, 126.33, 126.21, 125.84, 125.56, 125.20, 124.05, 123.79, 121.75, 121.04. HRMS (ESI) calcd. for $C_{15}H_9F_3$ $[M+H]^+$:247.0735, found 247.0739; $[M+Na]^+$: 269.0554, found 269.0558.

1-Fluorophenanthrene (y-11)

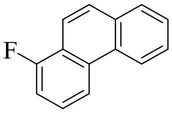

Following the general procedure on a 0.30 mmol scale, compound **y-11** (51.2 mg, 87 %) was obtained as white solid. **^{1}H NMR** (500 MHz, $CDCl_3$) δ 8.69 (d, *J* = 8.2 Hz, 1H), 8.49 (d, *J* = 8.4 Hz, 1H), 8.07 (d, *J* = 9.1 Hz, 1H), 7.95 (d, *J* = 7.6 Hz, 1H), 7.83 (d, *J* = 9.0 Hz, 1H), 7.73 – 7.69 (m, 1H), 7.68 – 7.65 (m, 1H), 7.61 (m, 1H), 7.32 (dd, *J* = 9.8, 7.6 Hz, 1H). **^{13}C NMR** (126 MHz, $CDCl_3$) δ 160.23, 158.24, 132.12,

132.08, 132.02, 129.59, 129.57, 128.73, 127.34, 127.32, 127.06, 126.95, 126.46, 126.39, 122.96, 121.57, 121.45, 118.49, 118.43, 118.36, 118.33, 111.04, 110.88. HRMS (ESI) calcd. for $C_{14}H_9F$ $[M+H]^+$:197.0767, found 197.0773; $[M+Na]^+$: 219.0586, found 219.0589.

1-Chlorophenanthrene (y-12)

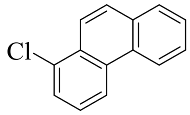

Following the general procedure on a 0.30 mmol scale, compound **y-12** (54.9 mg, 86 %) was obtained as white solid. **^{1}H NMR** (500 MHz, $CDCl_3$) δ 8.71 (d, *J* = 8.2 Hz, 1H), 8.66 (d, *J* = 8.4 Hz, 1H), 8.27 (d, *J* = 9.2 Hz, 1H), 7.96 (d, *J* = 7.7 Hz, 1H), 7.89 (d, *J* = 9.2 Hz, 1H), 7.72 (m, 2H), 7.69 – 7.65 (m, 1H), 7.61 – 7.57 (m, 1H). **^{13}C NMR** (126 MHz, $CDCl_3$) δ 132.70, 131.89, 131.88, 129.93, 129.32, 128.70, 128.21, 127.18, 127.07, 127.06, 126.43, 122.95, 122.50, 121.62. HRMS (ESI) calcd. for $C_{14}H_9Cl$ $[M+H]^+$:213.0471, found 213.0475; $[M+Na]^+$: 235.0290, found 235.0297.

1-Phenanthrenecarboxylic acid, methyl ester (y-13)

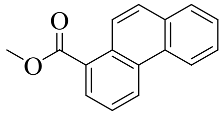

Following the general procedure on a 0.30 mmol scale, compound **y-13** (66.6 mg, 94 %) was obtained as light yellow oil. **^{1}H NMR** (500 MHz, $CDCl_3$) δ 8.93 (d, *J* = 8.4 Hz, 1H), 8.81 (d, *J* = 9.3 Hz, 1H), 8.71 (d, *J* = 8.2 Hz, 1H), 8.24 (dd, *J* = 7.4, 1.0 Hz, 1H), 7.97 – 7.93 (m, 1H), 7.90 (d, *J* = 9.3 Hz, 1H), 7.73 – 7.64 (m, 3H), 4.06 (s, 3H). **^{13}C NMR** (126 MHz, $CDCl_3$) δ 168.42, 131.61, 130.93, 130.63, 130.03, 129.81, 128.76, 128.53, 128.22, 127.10, 127.07, 126.90, 125.28, 123.74, 122.78, 52.30. HRMS (ESI) calcd. for $C_{16}H_{12}O_2$ $[M+H]^+$:237.0916, found 237.0912; $[M+Na]^+$: 259.0735, found 259.0731.

1,3-Dimethylphenanthrene (y-14)

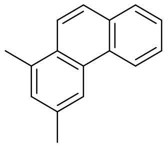

Following the general procedure on a 0.30 mmol scale, compound **y-14** (58.8 mg, 95 %) was obtained as white solid. **^{1}H NMR** (500 MHz, $CDCl_3$) δ 8.74 (d, *J* = 8.2 Hz, 1H), 8.43 (s, 1H), 7.94 (dd, *J* = 15.2, 8.0 Hz, 2H), 7.77 (d, *J* = 9.1 Hz, 1H), 7.68 (m, 1H), 7.65 – 7.60 (m, 1H), 7.34 (s, 1H), 2.77 (s, 3H), 2.63 (s, 3H). **^{13}C NMR** (126 MHz, $CDCl_3$) δ 135.73, 134.66, 131.84, 130.48, 130.38, 129.62, 128.75, 128.43, 126.30, 126.27, 125.72, 122.93, 122.77, 120.56, 22.06, 19.84. HRMS (ESI) calcd. for $C_{16}H_{14}$ $[M+H]^+$:207.1174, found 207.1179; $[M+Na]^+$: 229.0993, found 229.0997.

3-Fluoro-1-methylphenanthrene (y-15)

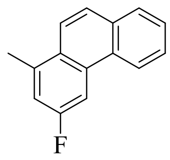

Following the general procedure on a 0.30 mmol scale, compound **y-15** (60.6 mg, 95 %) was obtained as white solid. **^{1}H NMR** (500 MHz, $CDCl_3$) δ 8.58 (d, J = 8.1 Hz, 1H), 8.21 (dd, J = 11.0, 2.2 Hz, 1H), 7.96 – 7.90 (m, 2H), 7.76 (d, J = 9.1 Hz, 1H), 7.70 – 7.63 (m, 2H), 7.25 (d, J = 9.1 Hz, 1H), 2.78 (s, 3H). **^{13}C NMR** (126 MHz, $CDCl_3$) δ 161.26, 158.83, 136.89, 136.80, 131.01, 130.94, 129.15, 129.10, 127.51, 126.54, 125.97, 125.55, 124.82, 124.79, 122.10, 121.39, 115.78, 115.54, 104.72, 104.51, 18.93. HRMS (ESI) calcd. for $C_{15}H_{11}F$ $[M+H]^+$:211.0923, found 211.0918; $[M+Na]^+$: 233.0742, found 233.0739.

7-Methoxy-1-methylphenanthrene (z-1)

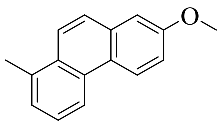

Following the general procedure on a 0.30 mmol scale, compound **z-1** (62.7 mg, 94 %) was obtained as white solid. **^{1}H NMR** (500 MHz, $CDCl_3$) δ 8.63 (d, J = 8.9 Hz, 1H), 8.51 (d, J = 8.4 Hz, 1H), 7.98 (d, J = 9.1 Hz, 1H), 7.75 (d, J = 9.1 Hz, 1H), 7.58 – 7.52 (m, 1H), 7.42 (d, J = 7.1 Hz, 1H), 7.31 (m, 2H), 4.00 (s, 3H), 2.78 (s, 3H). **^{13}C NMR** (126 MHz, $CDCl_3$) δ 158.20, 134.88, 133.07, 130.52, 129.82, 126.81, 126.25, 126.22, 125.07, 124.61, 123.49, 120.41, 117.20, 108.34, 55.41, 19.92. HRMS (ESI) calcd. for $C_{16}H_{14}O$ $[M+H]^+$:223.1123, found 223.1128; $[M+Na]^+$: 245.0942, found 245.0948.

1,7-Dimethylphenanthrene (z-2)

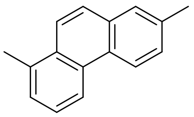

Following the general procedure on a 0.30 mmol scale, compound **z-2** (56.3 mg, 91 %) was obtained as white solid. **^{1}H NMR** (500 MHz, $CDCl_3$) δ 8.62 (d, J = 8.5 Hz, 1H), 8.58 (d, J = 8.3 Hz, 1H), 7.96 (d, J = 9.1 Hz, 1H), 7.75 (d, J = 9.1 Hz, 1H), 7.71 (s, 1H), 7.58 – 7.54 (m, 1H), 7.51 (dd, J = 8.5, 1.7 Hz, 1H), 7.45 (d, J = 7.0 Hz, 1H), 2.79 (s, 3H), 2.60 (s, 3H). **^{13}C NMR** (126 MHz, $CDCl_3$) δ 136.12, 134.81, 131.80, 130.45, 130.36, 128.51, 128.35, 127.98, 127.31, 126.42, 126.05, 122.85, 120.66, 21.45, 19.95. HRMS (ESI) calcd. for $C_{16}H_{14}$ $[M+H]^+$:207.1174, found 207.1179; $[M+Na]^+$: 229.0993, found 229.0999.

1-Methyl-7-(trifluoromethyl)phenanthrene (z-3)

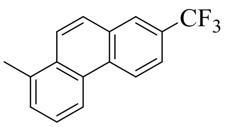

Following the general procedure on a 0.30 mmol scale, compound **z-3** (64.0 mg, 82 %) was obtained as white solid. **^{1}H NMR** (500 MHz, $CDCl_3$) δ 8.82 (d, J = 8.7 Hz, 1H), 8.62 (d, J = 8.3 Hz, 1H), 8.21 (s,

1H), 8.09 (d, *J* = 9.1 Hz, 1H), 7.86 (dd, *J* = 9.0, 2.9 Hz, 2H), 7.66 – 7.60 (m, 1H), 7.55 (d, *J* = 7.1 Hz, 1H), 2.81 (s, 3H). **^{13}C NMR** (126 MHz, $CDCl_3$) δ 135.22, 132.67, 131.51, 130.92, 129.69, 128.86, 126.76, 126.45, 125.82, 125.78, 124.37, 123.90, 122.36, 122.33, 121.23, 19.94. HRMS (ESI) calcd. for $C_{16}H_{11}F_3$[M+H]$^+$:261.0891, found 261.0886; [M+Na]$^+$: 283.0711, found 283.0708.

7-Fluoro-1-methylphenanthrene (z-4)

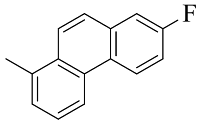

Following the general procedure on a 0.30 mmol scale, compound **z-4** (53.6 mg, 85 %) was obtained as white solid. **^{1}H NMR** (500 MHz, $CDCl_3$) δ 8.69 (dd, *J* = 9.1, 5.4 Hz, 1H), 8.53 (d, *J* = 8.4 Hz, 1H), 8.01 (d, *J* = 9.1 Hz, 1H), 7.74 (d, *J* = 9.1 Hz, 1H), 7.62 – 7.53 (m, 2H), 7.48 (d, *J* = 7.1 Hz, 1H), 7.42 (m, 1H), 2.79 (s, 3H). **^{13}C NMR** (126 MHz, $CDCl_3$) δ 162.21, 160.25, 135.08, 133.05, 132.98, 130.30, 130.29 , 130.18, 127.61, 127.29, 127.27, 126.58, 125.91, 125.88, 125.38, 125.31, 124.20, 120.67, 115.61, 115.42, 112.43, 112.27, 19.95. HRMS (ESI) calcd. for $C_{15}H_{11}F$ [M+H]$^+$:211.0923, found 211.0928; [M+Na]$^+$: 233.0742, found 233.0749.

7-Chloro-1-methylphenanthrene (z-5)

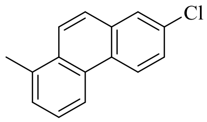

Following the general procedure on a 0.30 mmol scale, compound **z-5** (59.2 mg, 87 %) was obtained as white solid. **^{1}H NMR** (500 MHz, $CDCl_3$) δ 8.63 (d, *J* = 8.9 Hz, 1H), 8.53 (d, *J* = 8.4 Hz, 1H), 8.00 (d, *J* = 9.1 Hz, 1H), 7.89 (d, *J* = 2.2 Hz, 1H), 7.72 – 7.69 (m, 1H), 7.63 – 7.57 (m, 2H), 7.49 (d, *J* = 7.1 Hz, 1H), 2.78 (s, 3H). **^{13}C NMR** (126 MHz, $CDCl_3$) δ 135.10, 132.68, 132.20, 130.67, 129.97, 128.99, 128.05, 127.34, 126.99, 126.60, 125.59, 124.67, 124.19, 120.76, 19.93. HRMS (ESI) calcd. for $C_{15}H_{11}Cl$ [M+H]$^+$:227.0628, found 227.0623; [M+Na]$^+$: 249.0447, found 249.0443.

6,7-Dimethoxy-1-methylphenanthrene (z-6)

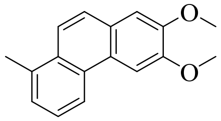

Following the general procedure on a 0.30 mmol scale, compound **z-6** (71.9 mg, 95 %) was obtained as white solid. **^{1}H NMR** (500 MHz, $CDCl_3$) δ 8.44 (d, *J* = 8.4 Hz, 1H), 8.03 (s, 1H), 7.87 (d, *J* = 9.0 Hz, 1H), 7.70 (d, *J* = 9.0 Hz, 1H), 7.58 – 7.48 (m, 1H), 7.40 (d, *J* = 7.0 Hz, 1H), 7.25 (s, 1H), 4.12 (s, 3H), 4.06 (s, 3H), 2.76 (s, 3H). **^{13}C NMR** (126 MHz, $CDCl_3$) δ 149.32, 149.25, 134.96, 130.14, 129.74, 126.83, 126.74, 125.76, 125.73, 125.26, 121.19, 120.41, 108.09, 103.63, 55.99, 55.91, 20.01. HRMS (ESI) calcd. for $C_{17}H_{16}O_2$ [M+H]$^+$:253.1229, found 253.1224; [M+Na]$^+$: 275.1048, found 275.1043.

2. ^{1}H NMR and ^{13}C NMR spectra for the products

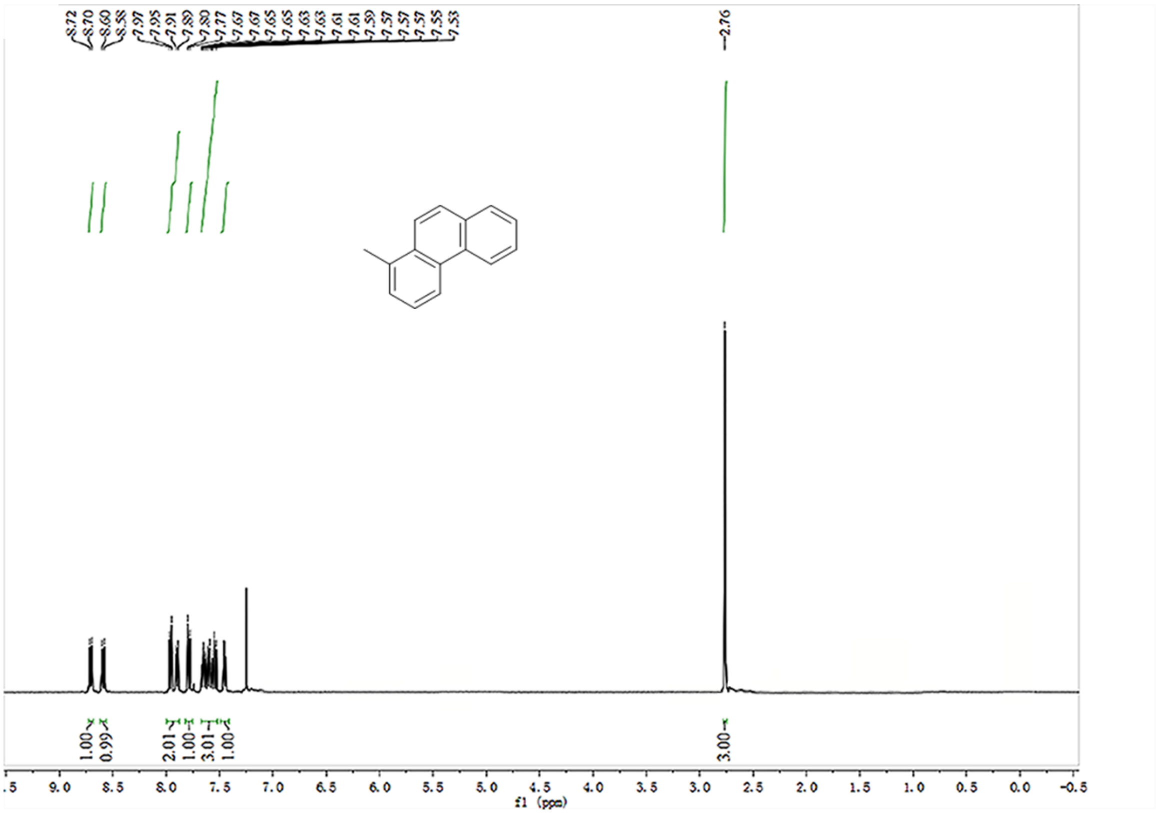

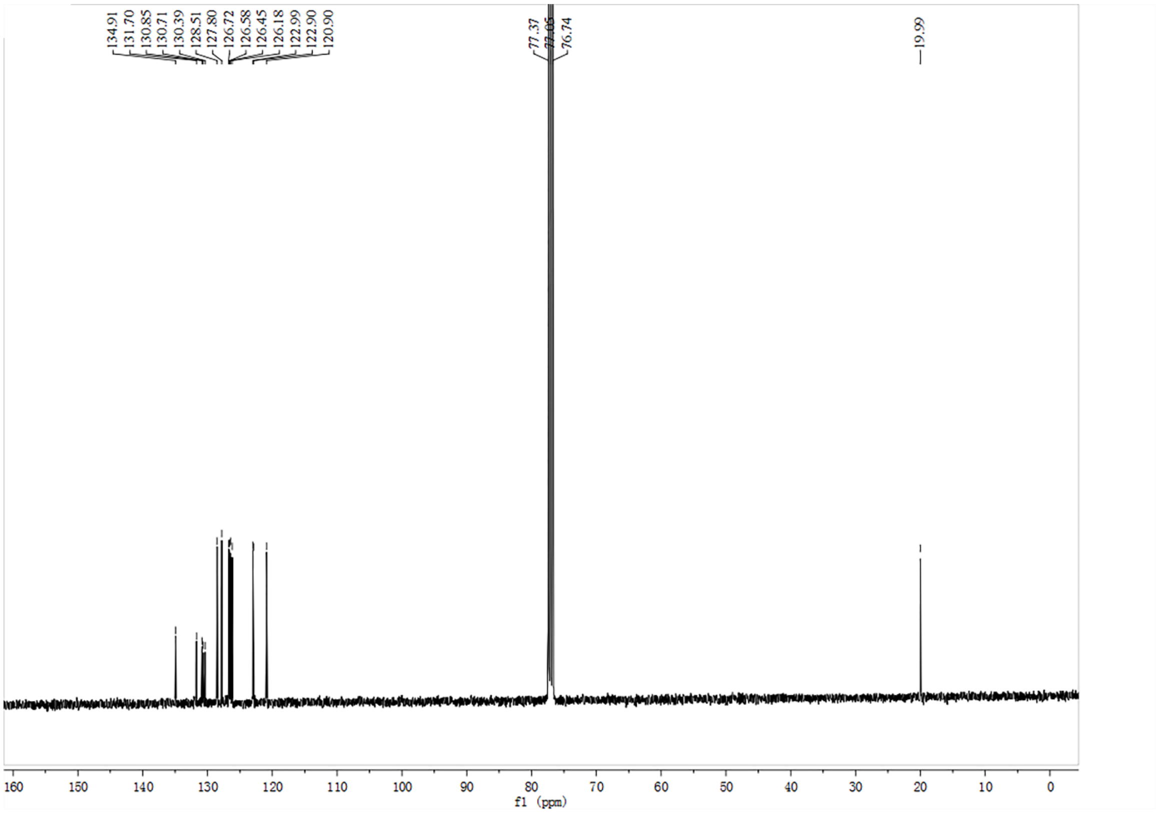

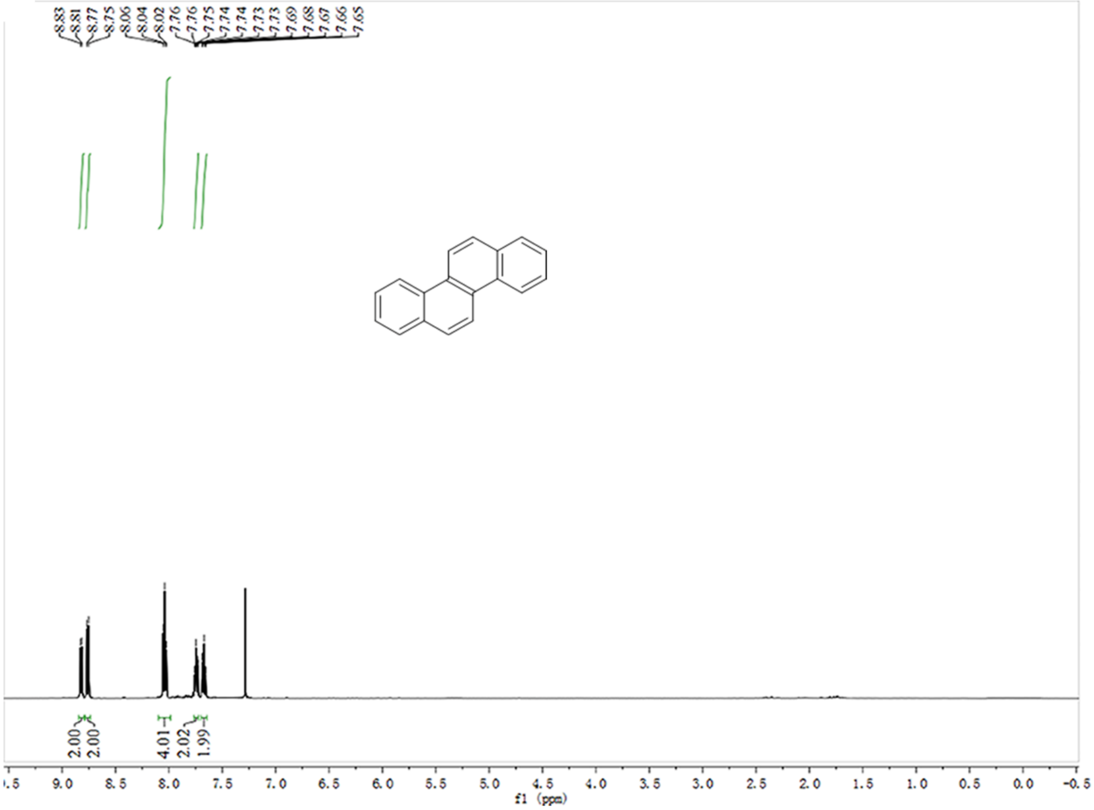
8.83
8.81
8.77
8.75
8.06
8.04
8.02
7.76
7.76
7.75
7.74
7.74
7.73
7.73
7.69
7.68
7.67
7.66
7.65
2.00
2.00
4.01
2.02
1.99
9.0 8.5 8.0 7.5 7.0 6.5 6.0 5.5 5.0 4.5 4.0 3.5 3.0 2.5 2.0 1.5 1.0 0.5 0.0 -0.5
f1 (ppm)

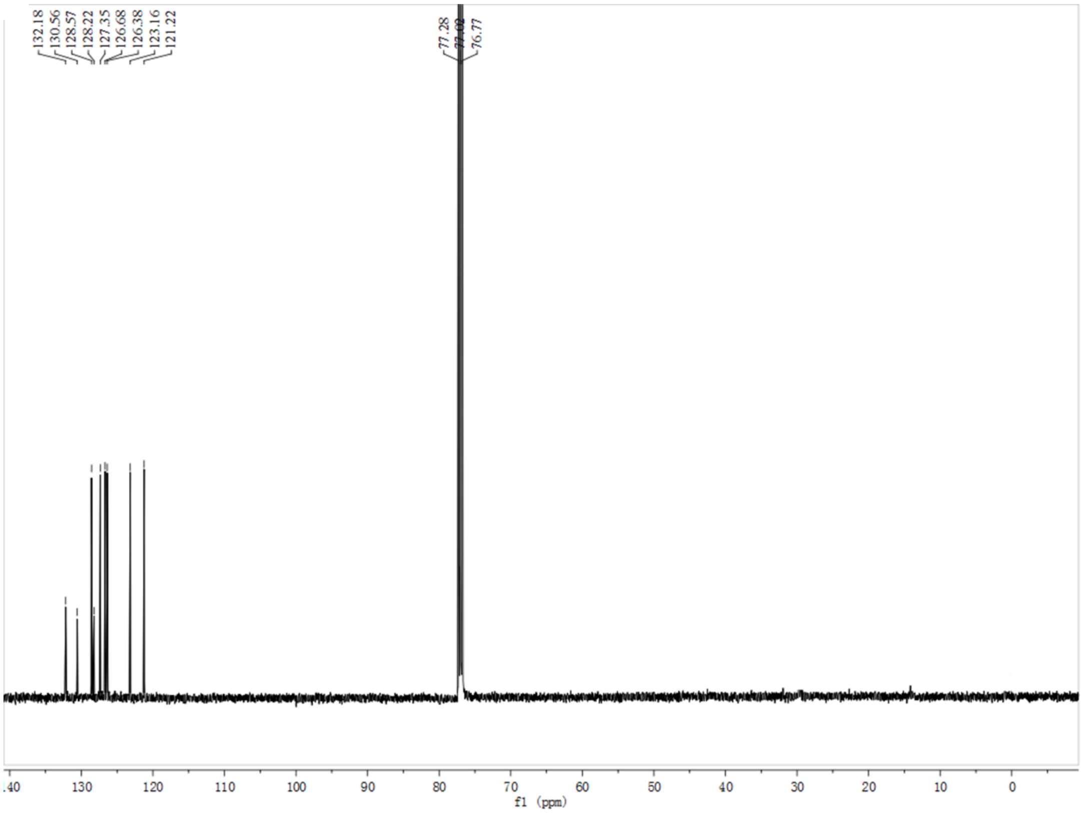
132.18
130.56
128.57
128.22
127.35
126.68
126.38
123.16
121.22
77.28
77.02
76.77
130 120 110 100 90 80 70 60 50 40 30 20 10 0
f1 (ppm)

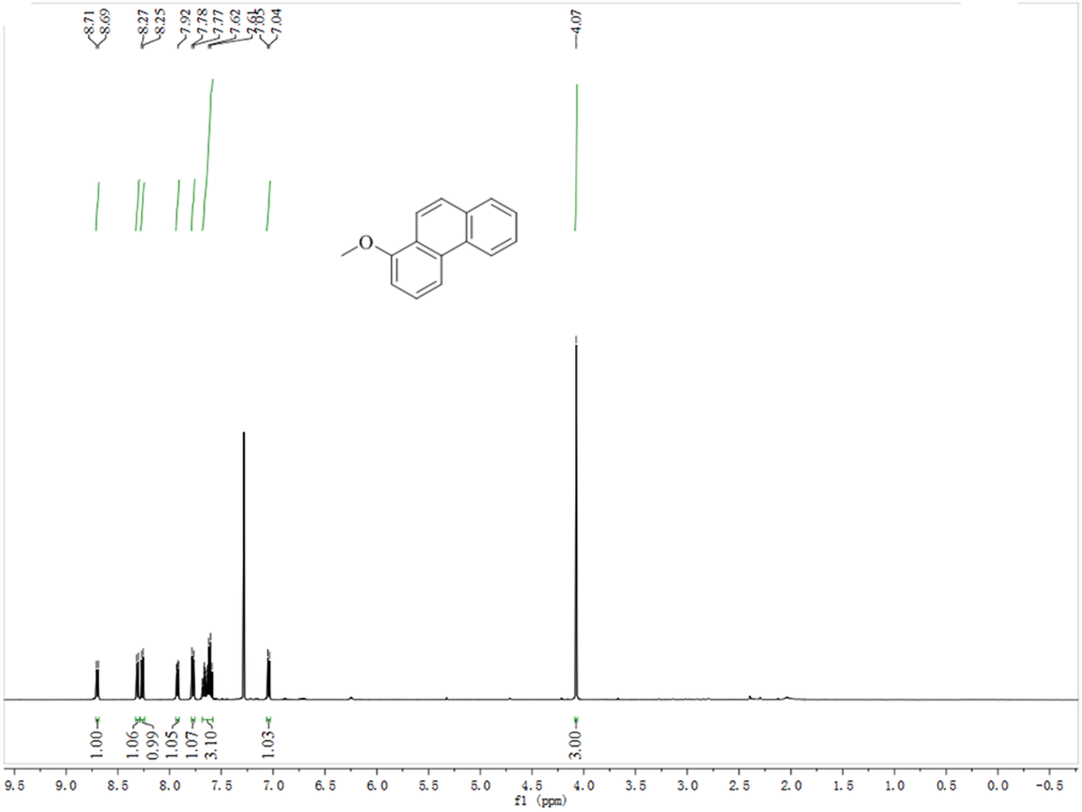
8.71
8.69
8.27
8.25
7.92
7.78
7.77
7.62
7.61
7.05
7.04
4.07
O
1.00
1.06
0.99
1.05
1.07
3.10
1.03
3.00
9.5 9.0 8.5 8.0 7.5 7.0 6.5 6.0 5.5 5.0 4.5 4.0 3.5 3.0 2.5 2.0 1.5 1.0 0.5 0.0 -0.5
f1 (ppm)

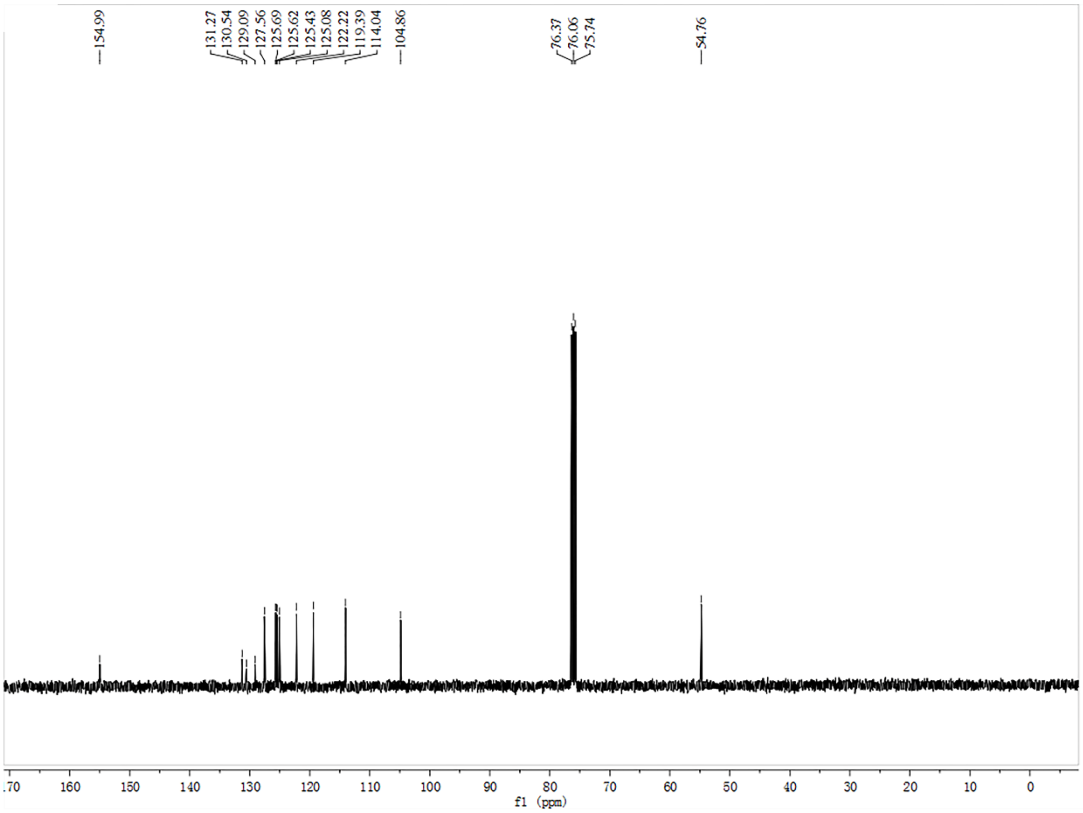
154.99
131.27
130.54
129.09
127.56
125.69
125.62
125.43
125.08
122.22
119.39
114.04
104.86
76.37
76.06
75.74
54.76
170 160 150 140 130 120 110 100 90 80 70 60 50 40 30 20 10 0
f1 (ppm)

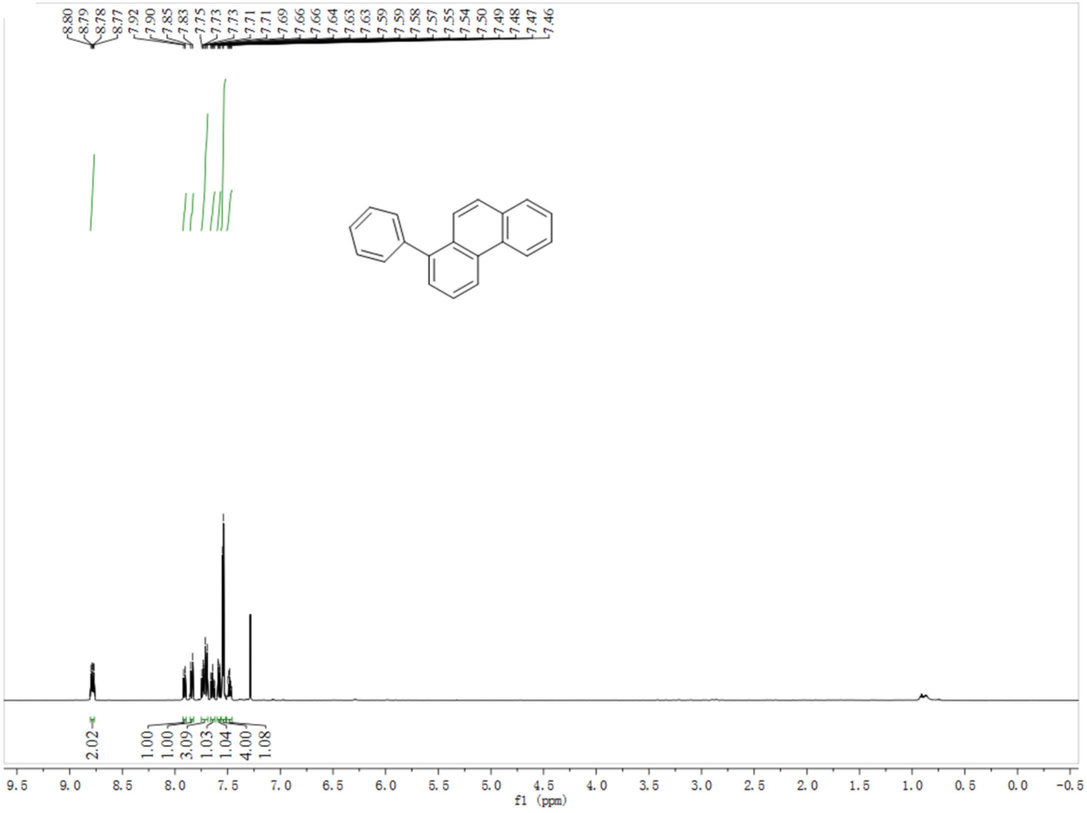
8.80
8.79
8.78
8.77
7.92
7.90
7.85
7.83
7.75
7.73
7.73
7.71
7.71
7.69
7.66
7.66
7.64
7.63
7.63
7.59
7.59
7.58
7.57
7.55
7.54
7.50
7.49
7.48
7.47
7.46
2.02
1.00
1.00
3.09
1.03
1.04
4.00
1.08
f1 (ppm)

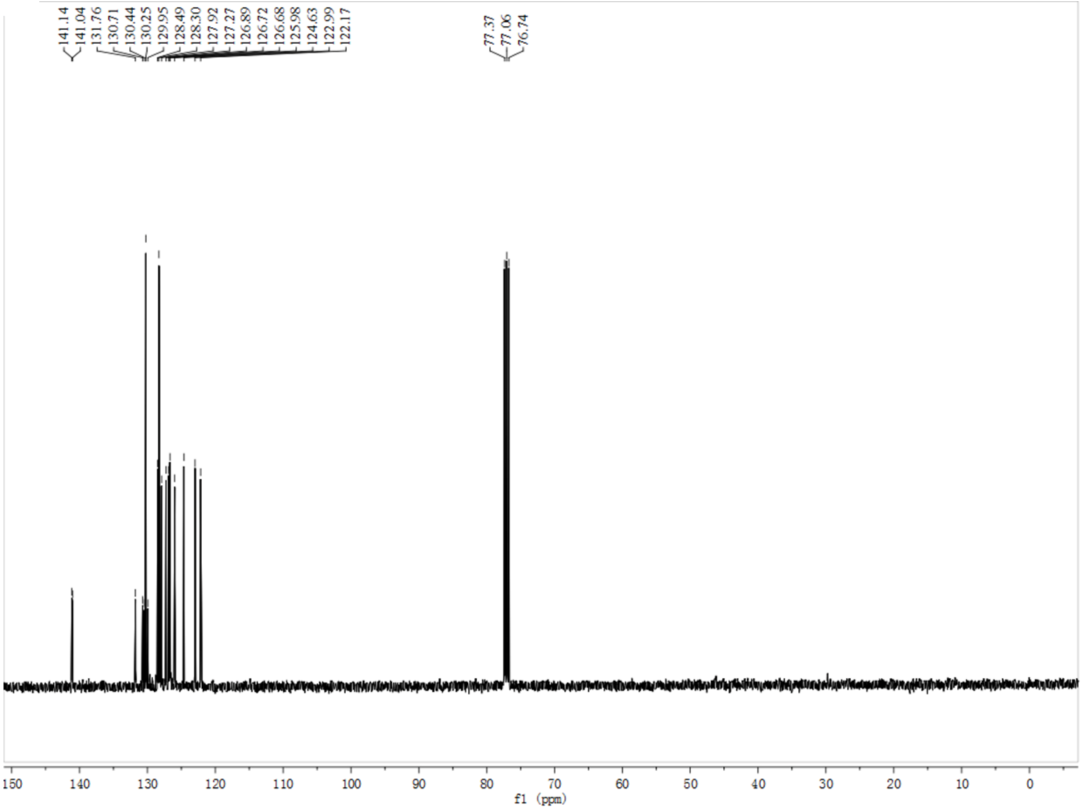
141.14
141.04
131.76
130.71
130.44
130.25
129.95
128.49
128.30
127.92
127.27
126.89
126.72
126.68
125.98
124.63
122.99
122.17
77.37
77.06
76.74
f1 (ppm)

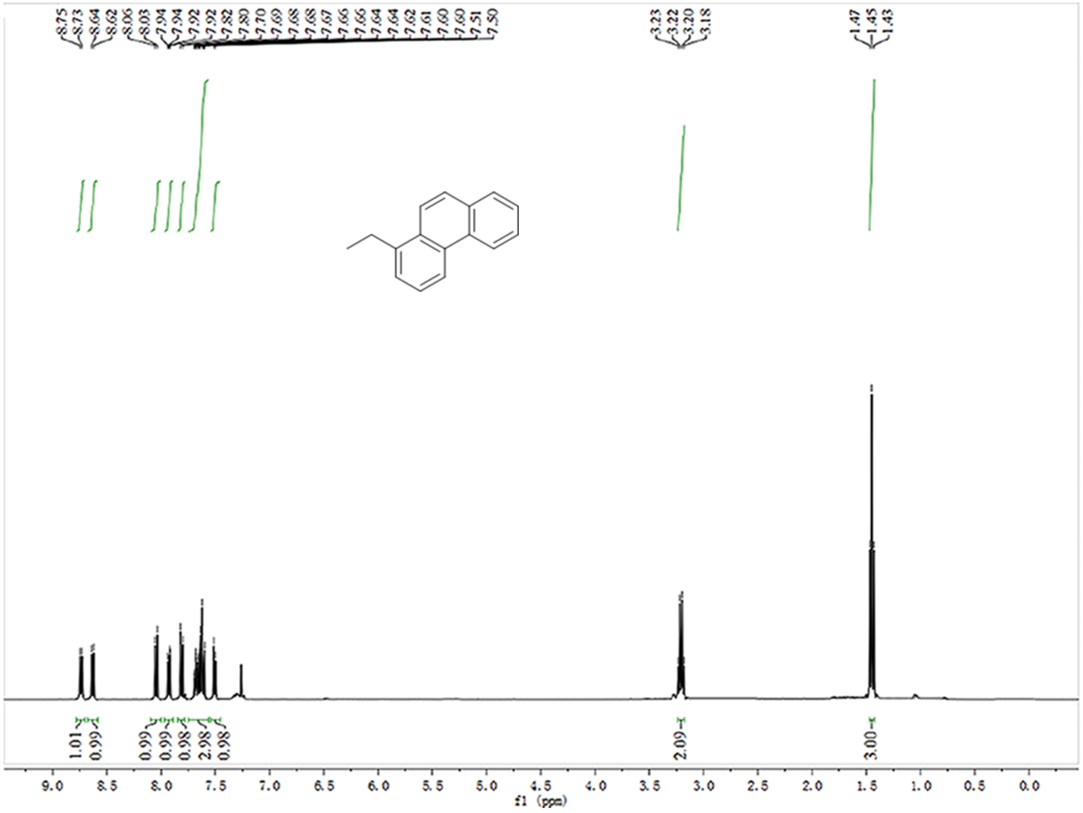

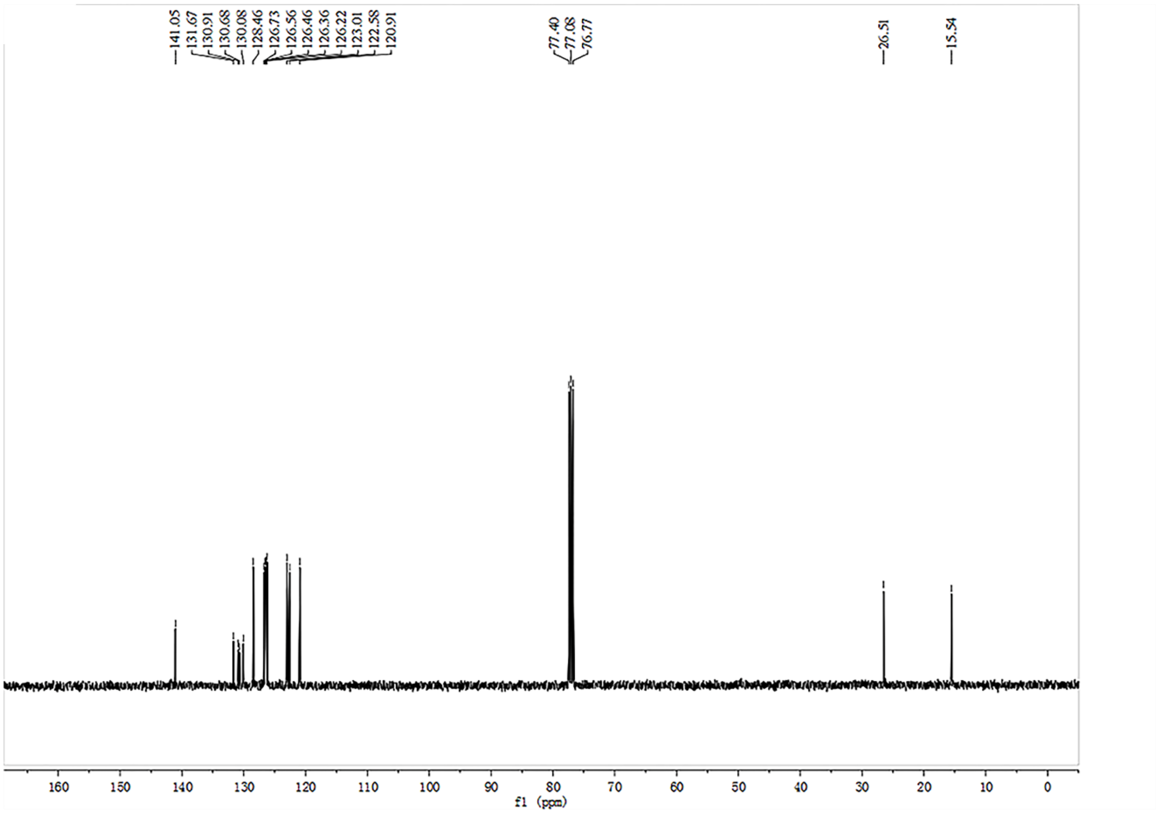

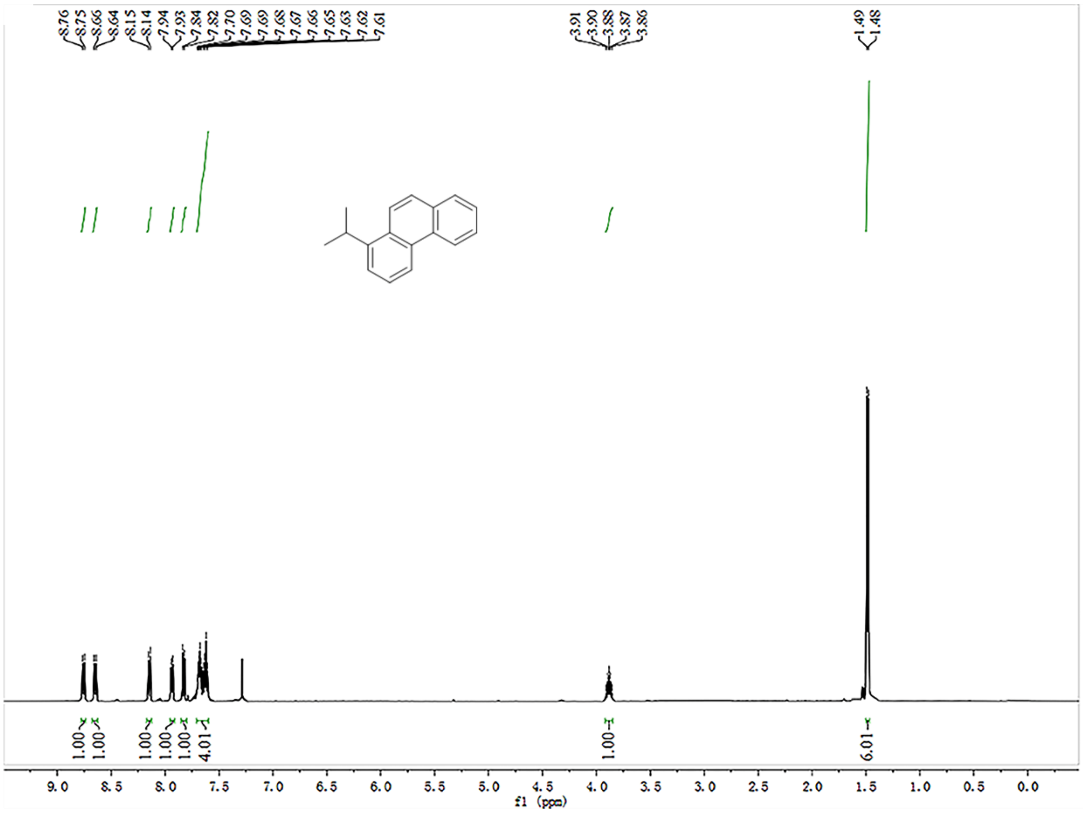

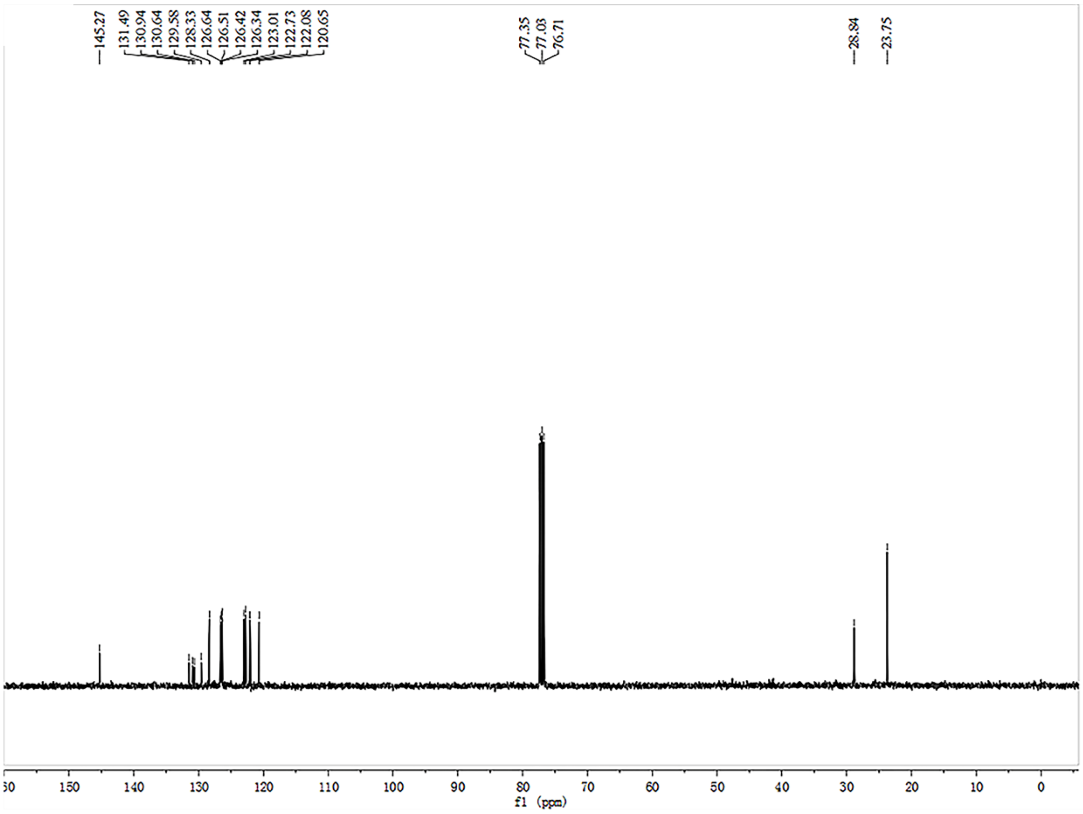

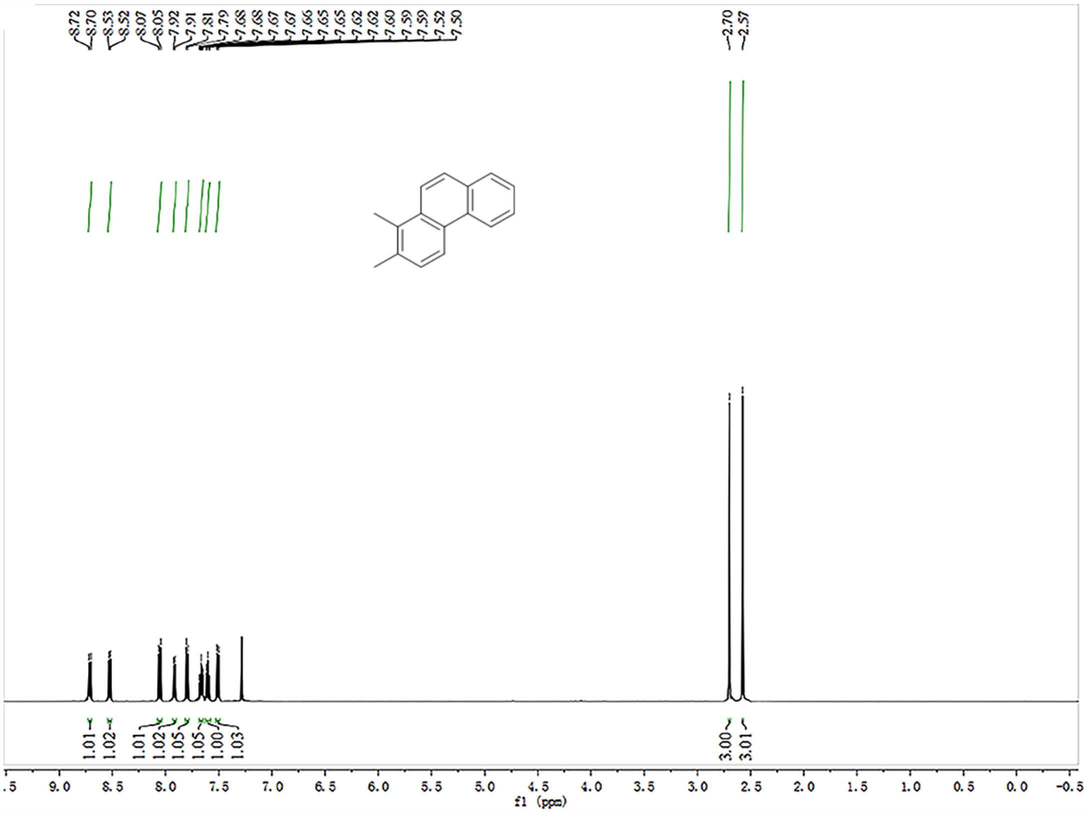
8.72
8.70
8.53
8.52
8.07
8.05
7.92
7.91
7.81
7.79
7.68
7.68
7.67
7.67
7.66
7.65
7.65
7.62
7.62
7.60
7.59
7.59
7.52
7.50
2.70
2.57
1.01
1.02
1.01
1.02
1.05
1.05
1.00
1.03
3.00
3.01
f1 (ppm)

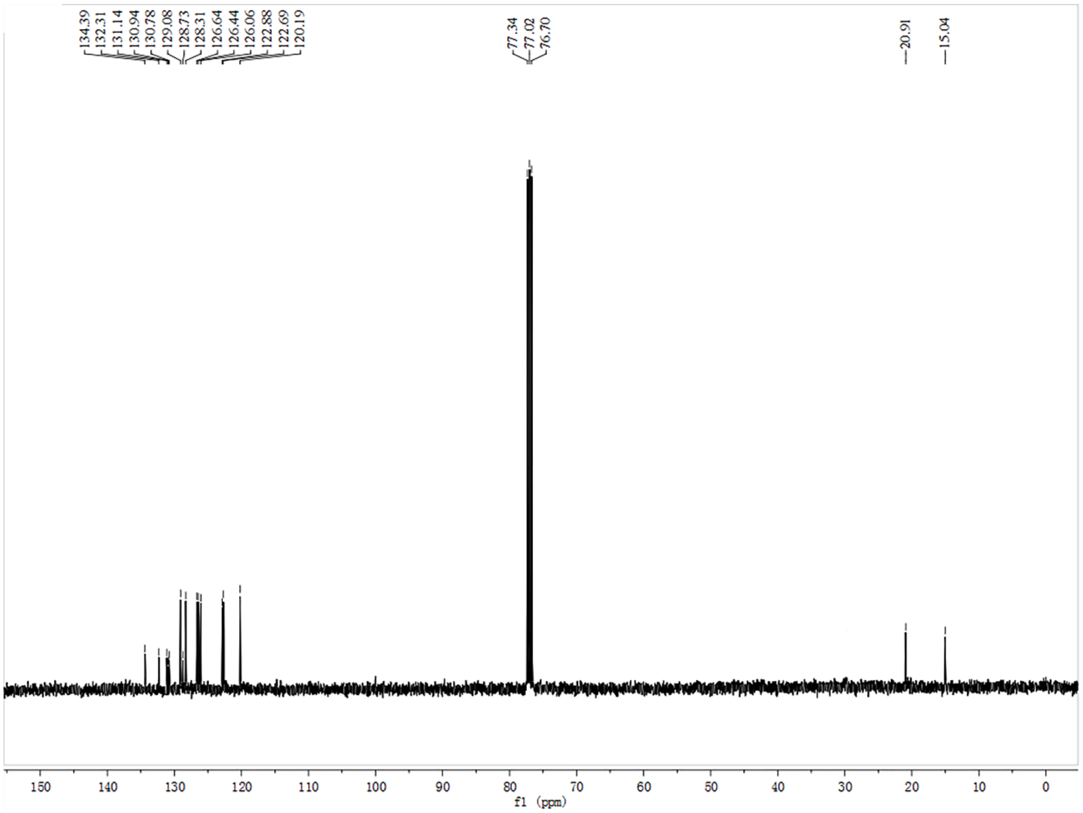
134.39
132.31
131.14
130.94
130.78
129.08
128.73
128.31
126.64
126.44
126.06
122.88
122.69
120.19
77.34
77.02
76.70
20.91
15.04
f1 (ppm)

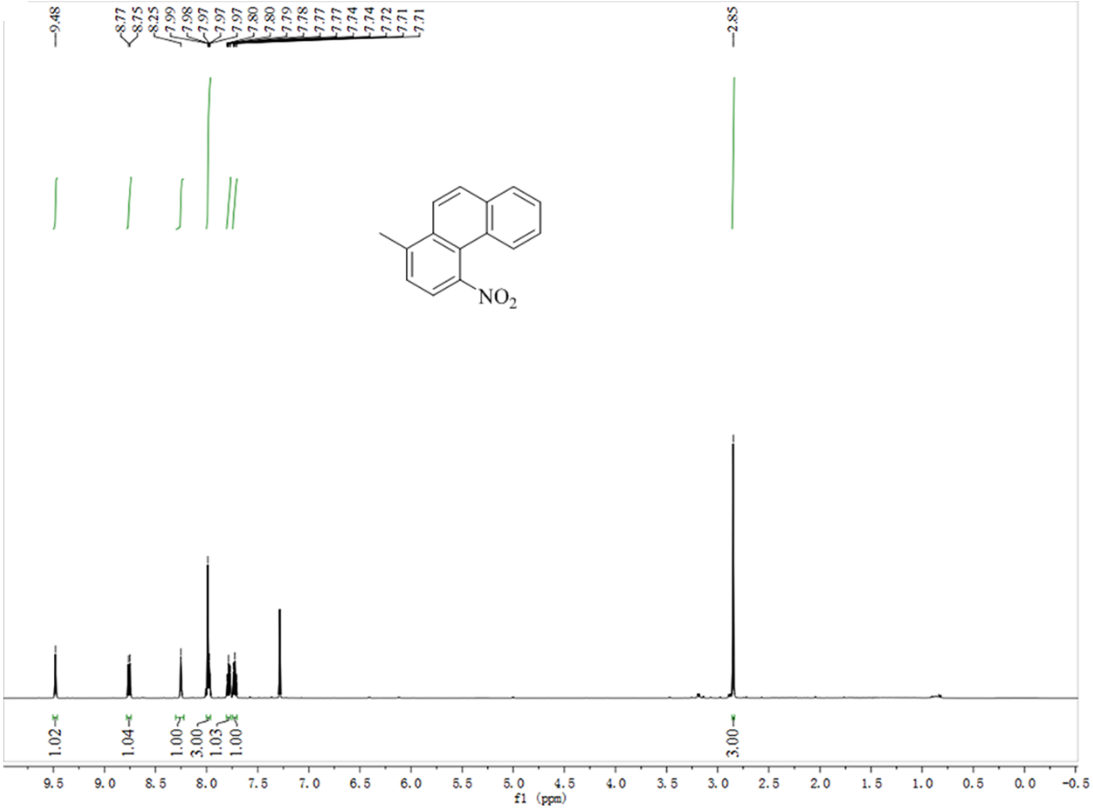
9.48
8.77
8.75
8.25
7.99
7.98
7.97
7.97
7.97
7.80
7.80
7.79
7.78
7.77
7.77
7.74
7.74
7.72
7.71
7.71
2.85
NO2
1.02
1.04
1.00
3.00
1.03
1.00
3.00
f1 (ppm)

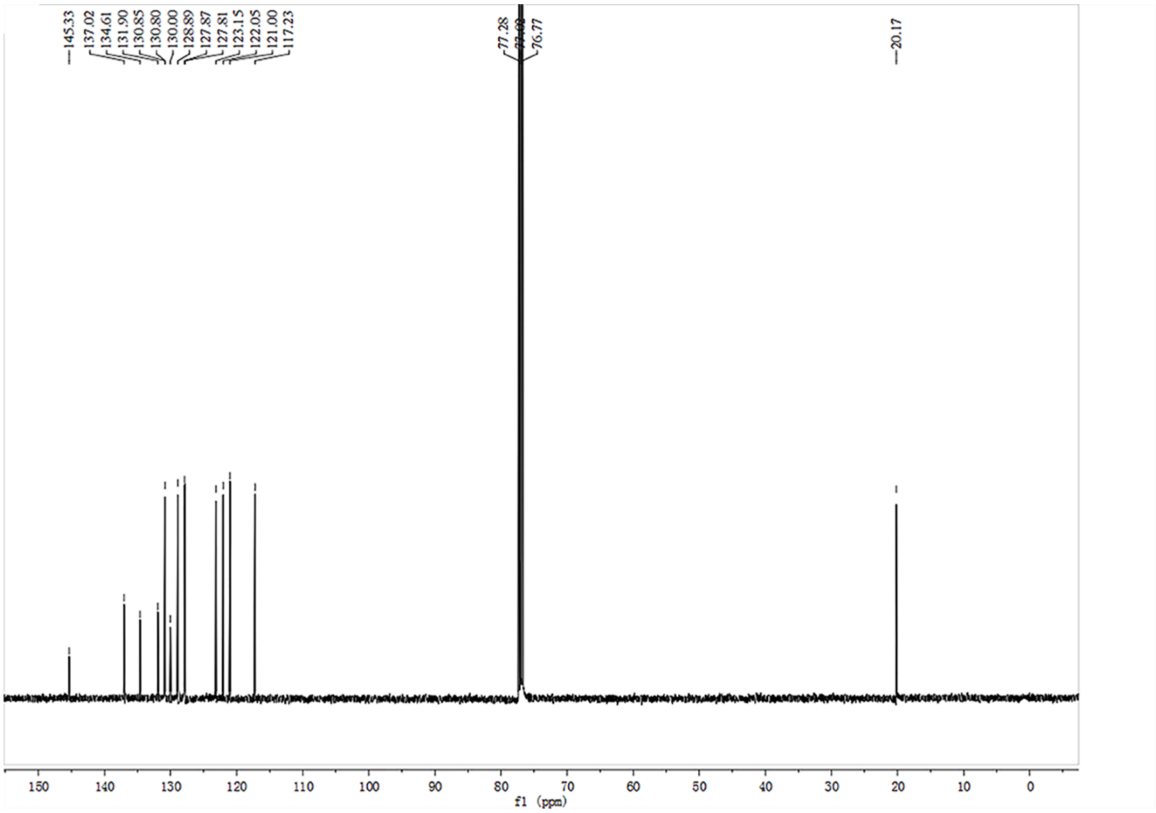
145.33
137.02
134.61
131.90
130.85
130.80
130.00
128.89
127.87
127.81
123.15
122.05
121.00
117.23
77.28
76.77
20.17
f1 (ppm)

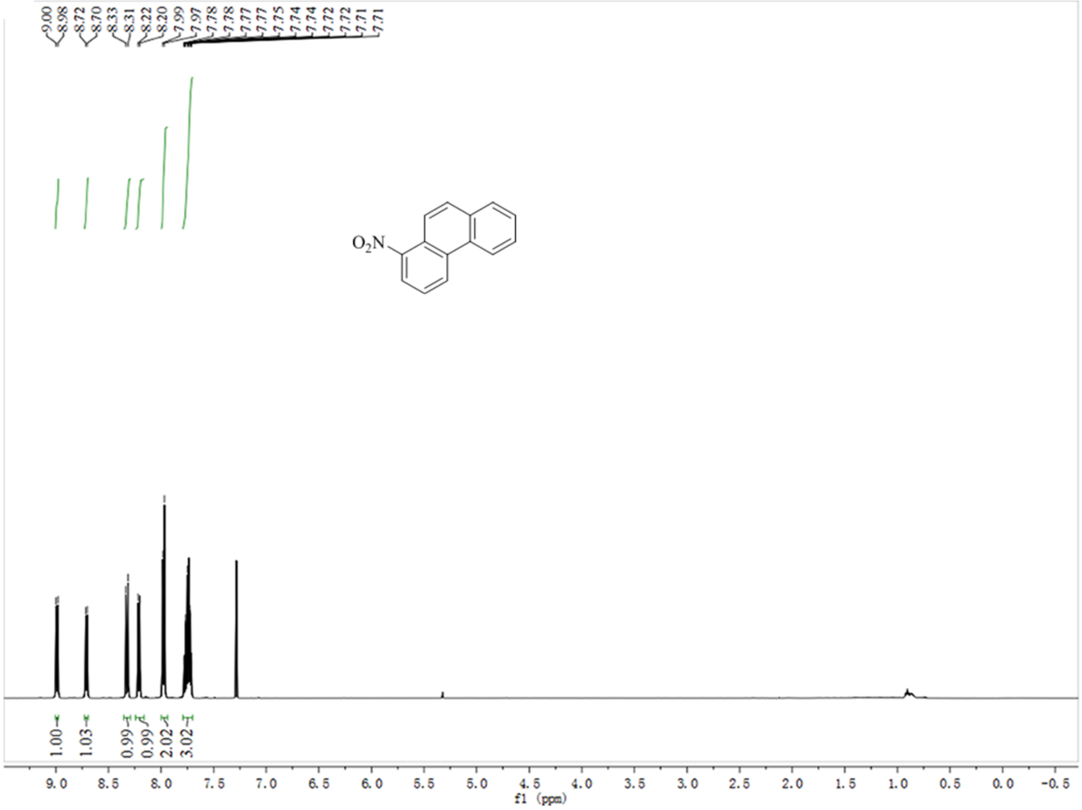
9.00
8.98
8.72
8.70
8.33
8.31
8.22
8.20
7.99
7.97
7.78
7.78
7.77
7.77
7.75
7.74
7.74
7.72
7.72
7.71
7.71
O_2N
1.00
1.03
0.99
0.99
2.02
3.02
f1 (ppm)

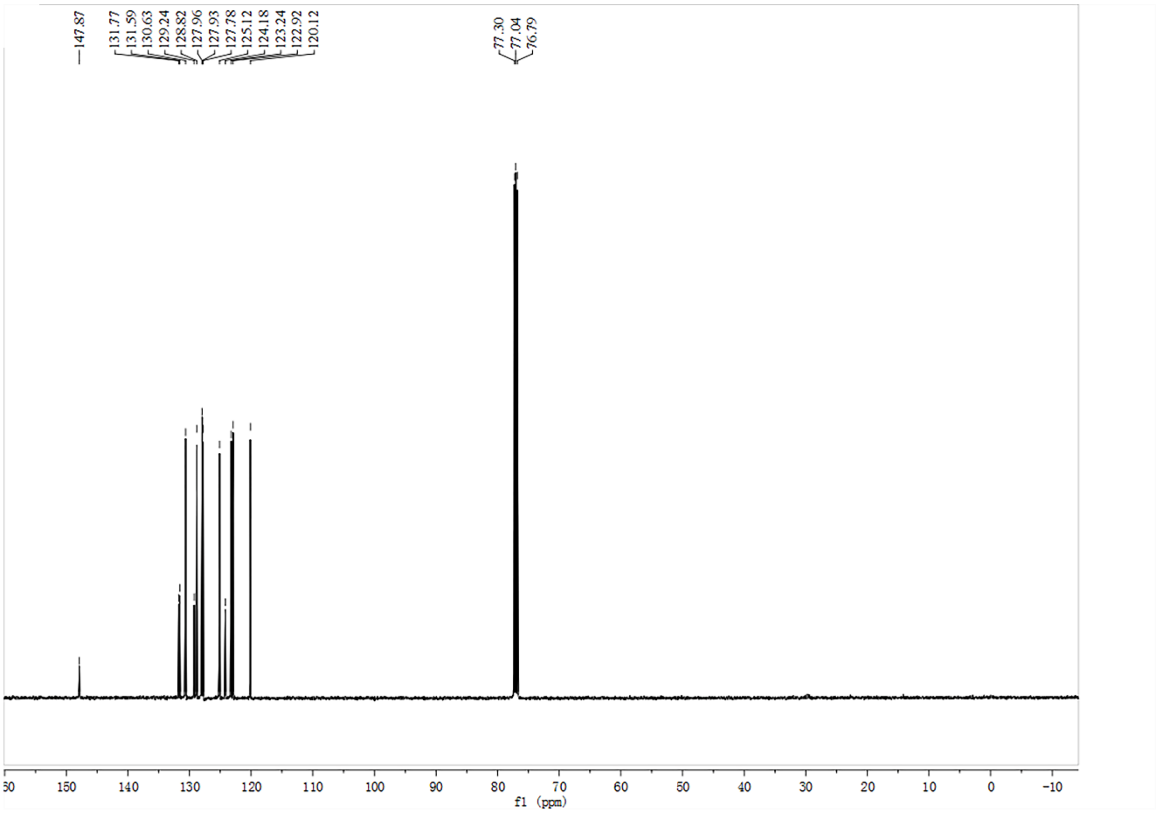
147.87
131.77
131.59
130.63
129.24
128.82
127.96
127.93
127.78
125.12
124.18
123.24
122.92
120.12
77.30
77.04
76.79
f1 (ppm)

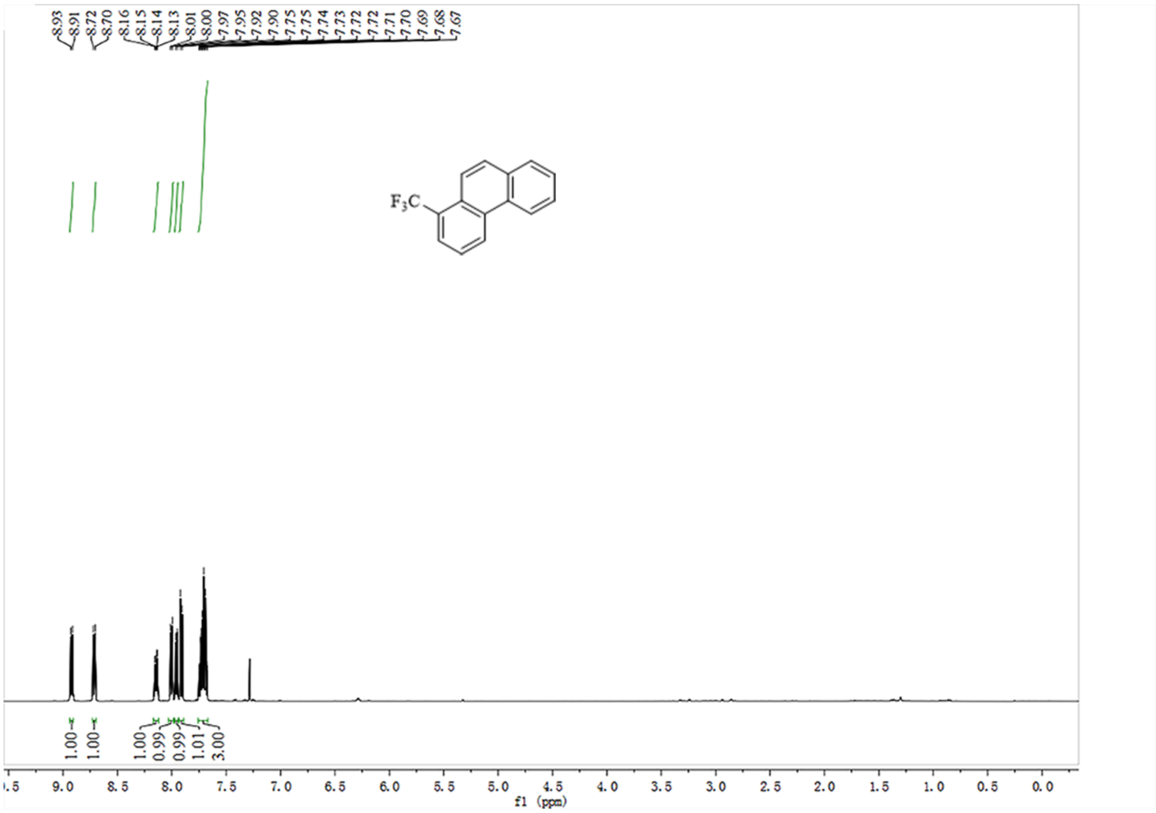
8.93
8.91
8.72
8.70
8.16
8.15
8.14
8.13
8.01
8.00
7.97
7.95
7.92
7.90
7.75
7.75
7.74
7.73
7.72
7.72
7.71
7.70
7.69
7.68
7.67
F3C
1.00
1.00
1.00
0.99
0.99
1.01
3.00
f1 (ppm)

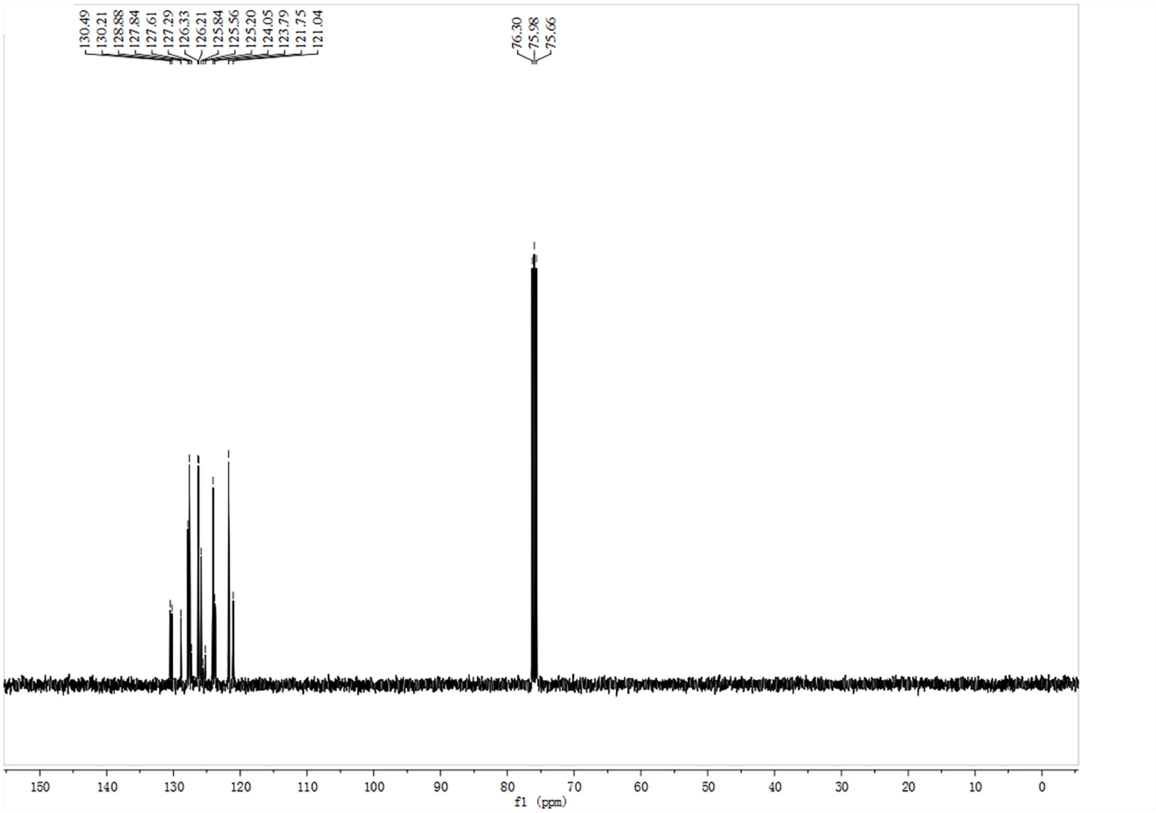
130.49
130.21
128.88
127.84
127.61
127.29
126.33
126.21
125.84
125.56
125.20
124.05
123.79
121.75
121.04
76.30
75.98
75.66
f1 (ppm)

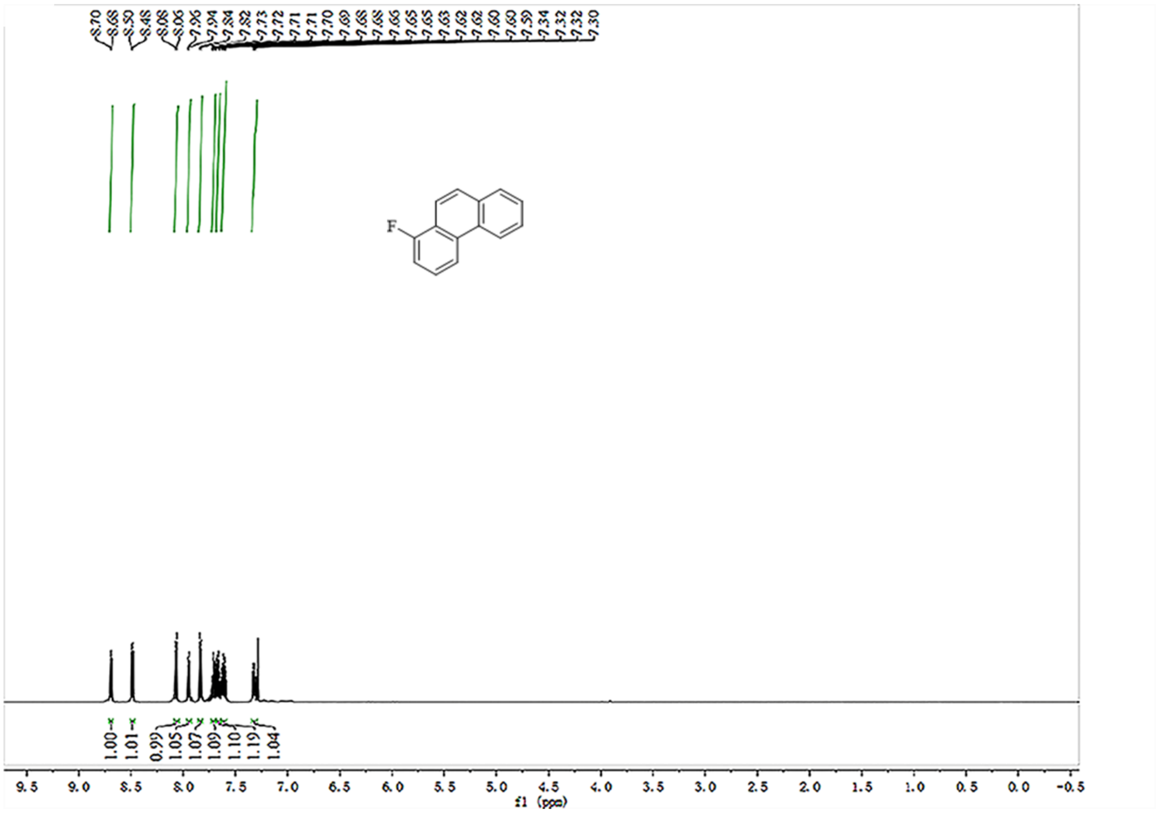

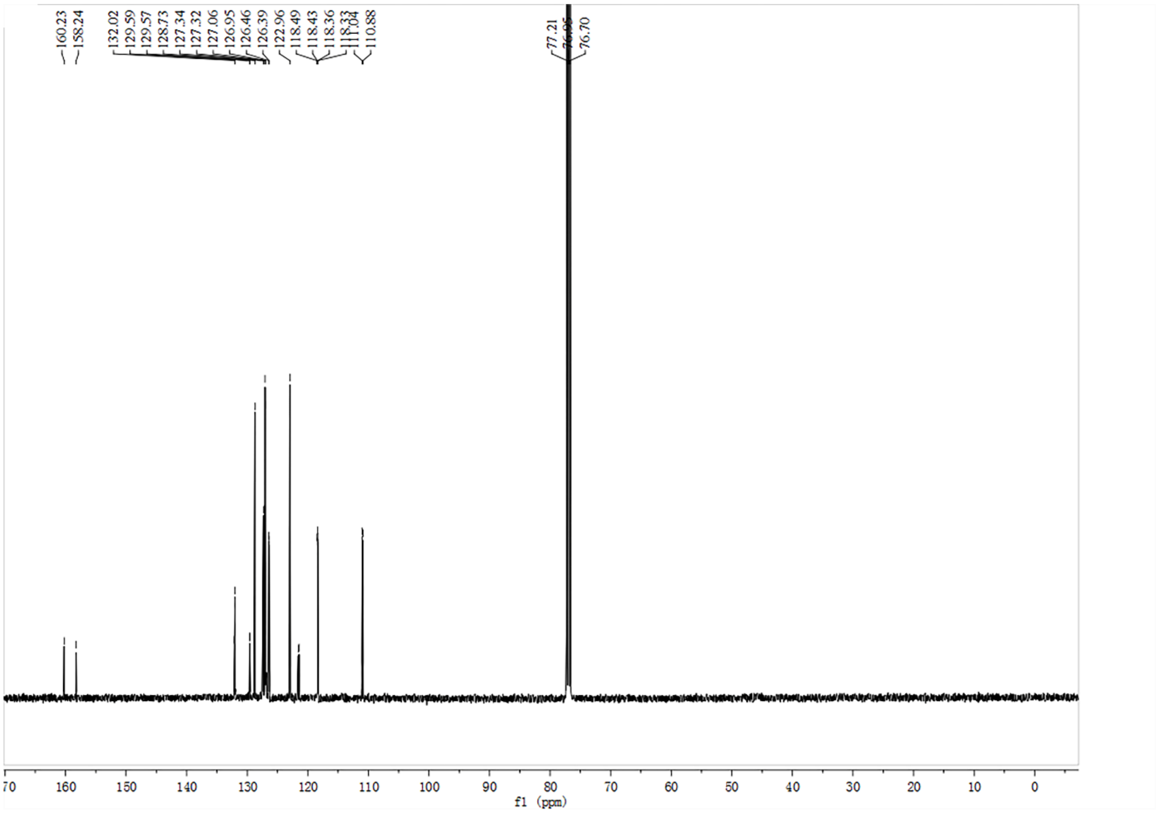

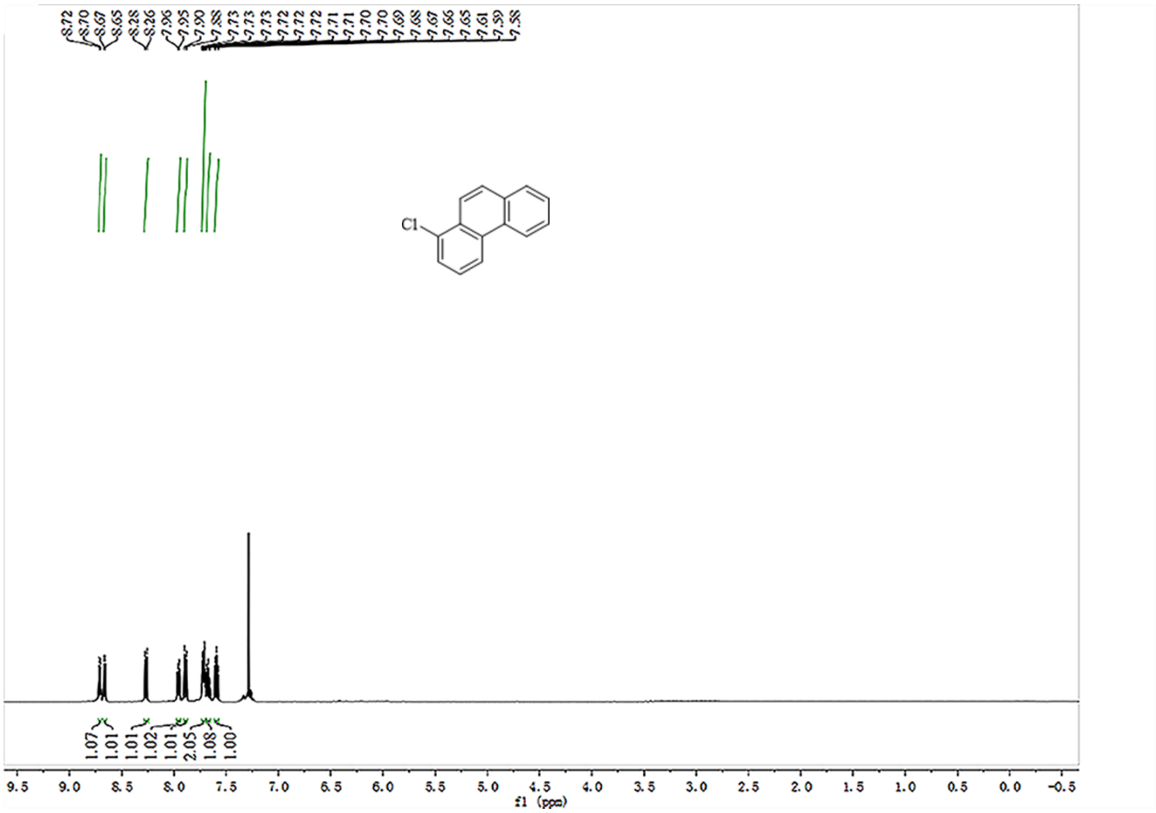

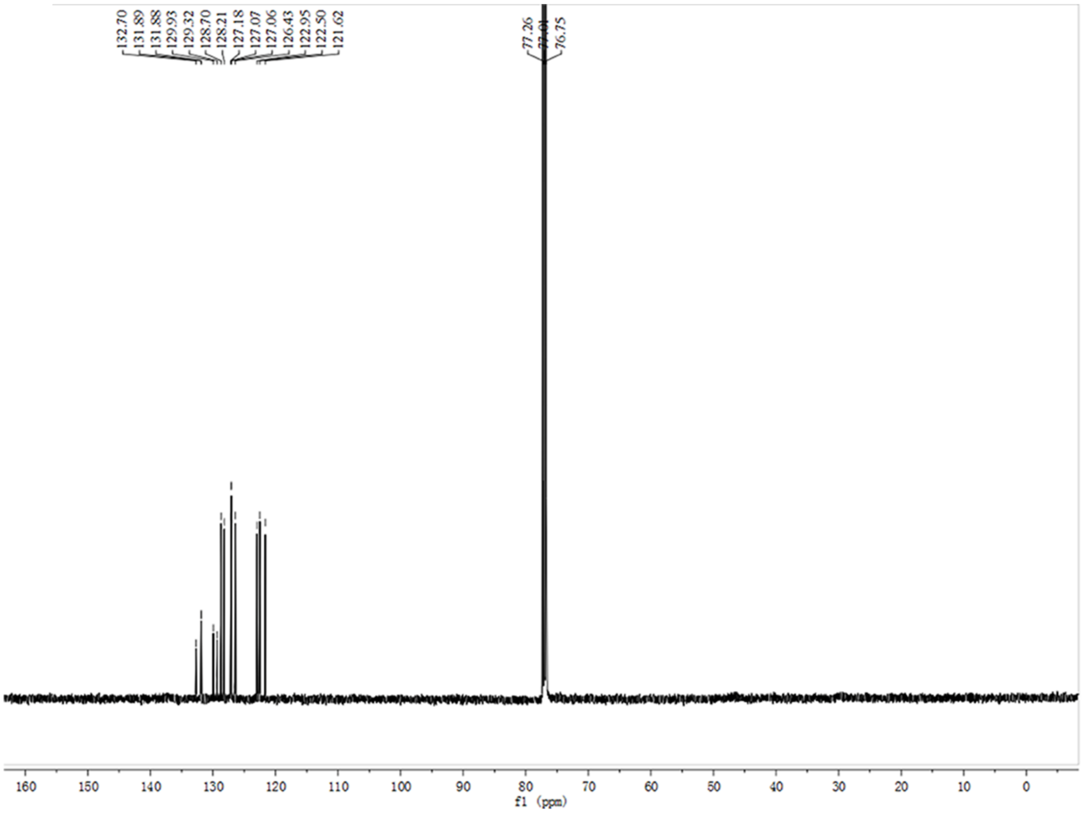

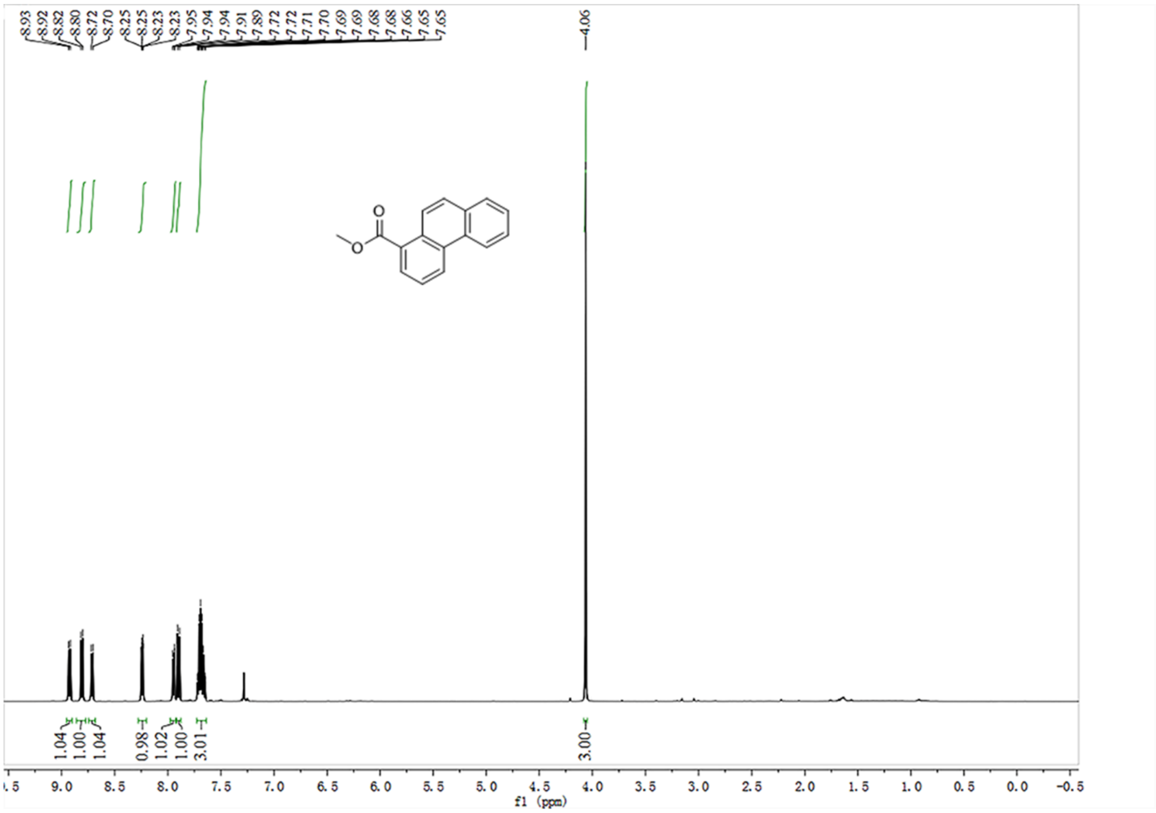

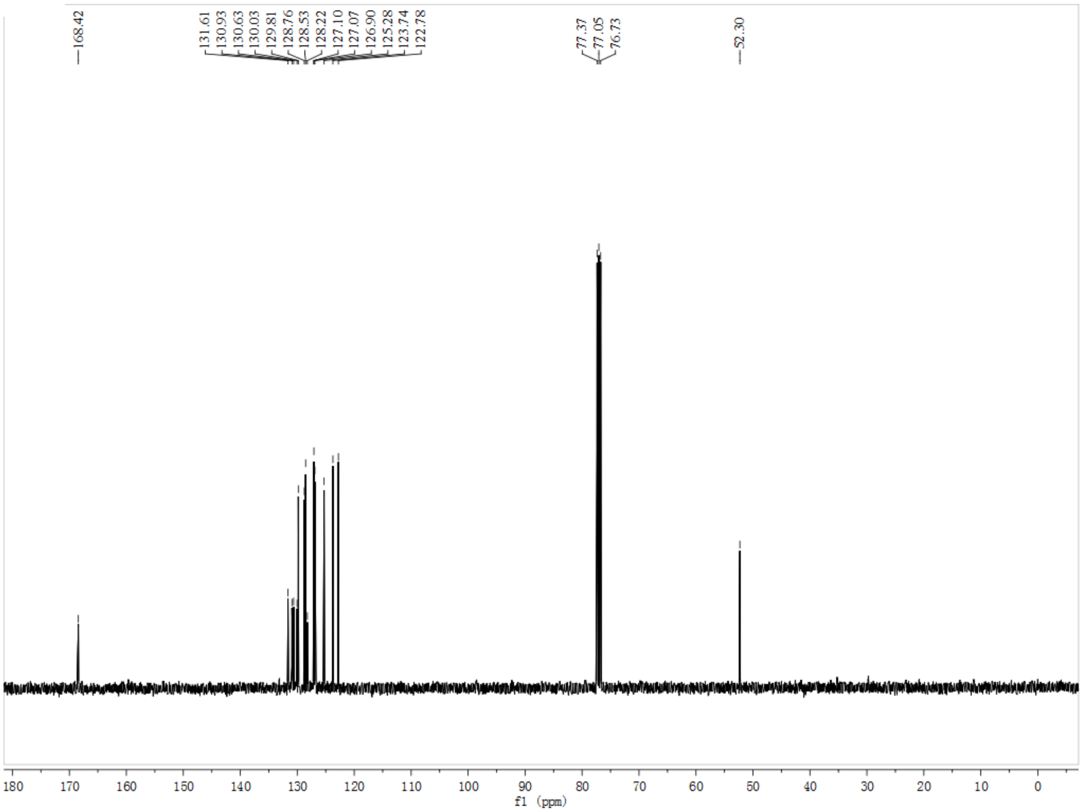

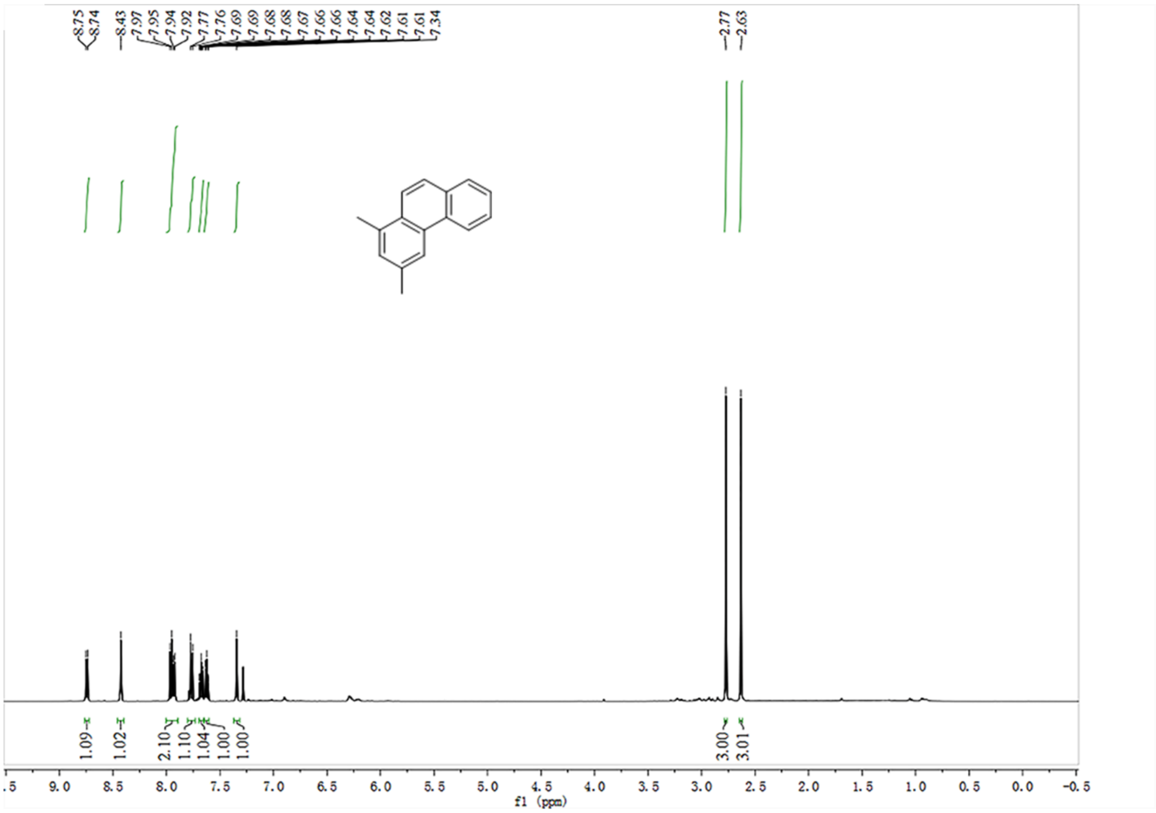
8.75
8.74
8.43
7.97
7.95
7.94
7.92
7.77
7.76
7.69
7.69
7.68
7.68
7.67
7.66
7.66
7.64
7.64
7.62
7.61
7.61
7.34
2.77
2.63
1.09
1.02
2.10
1.10
1.04
1.00
1.00
3.00
3.01
f1 (ppm)

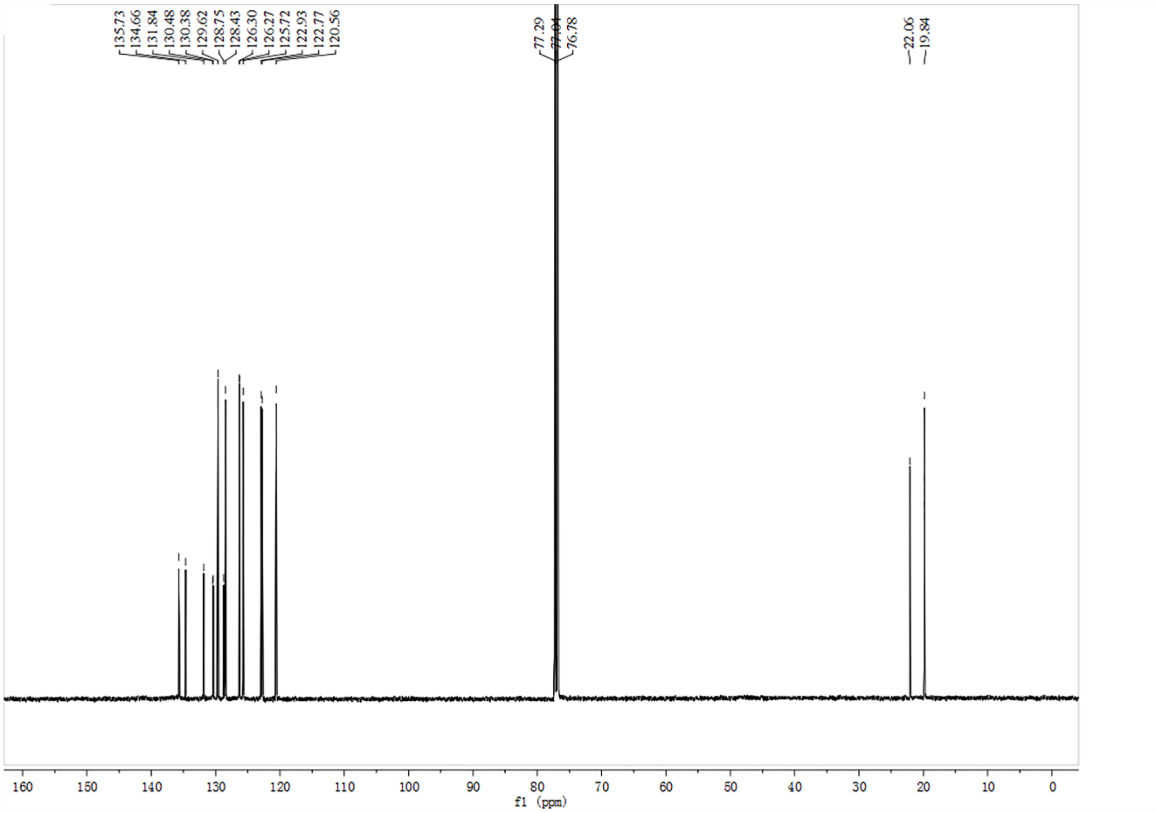
135.73
134.66
131.84
130.48
130.38
129.62
128.75
128.43
126.30
126.27
125.72
122.93
122.77
120.56
77.29
77.04
76.78
22.06
19.84
f1 (ppm)

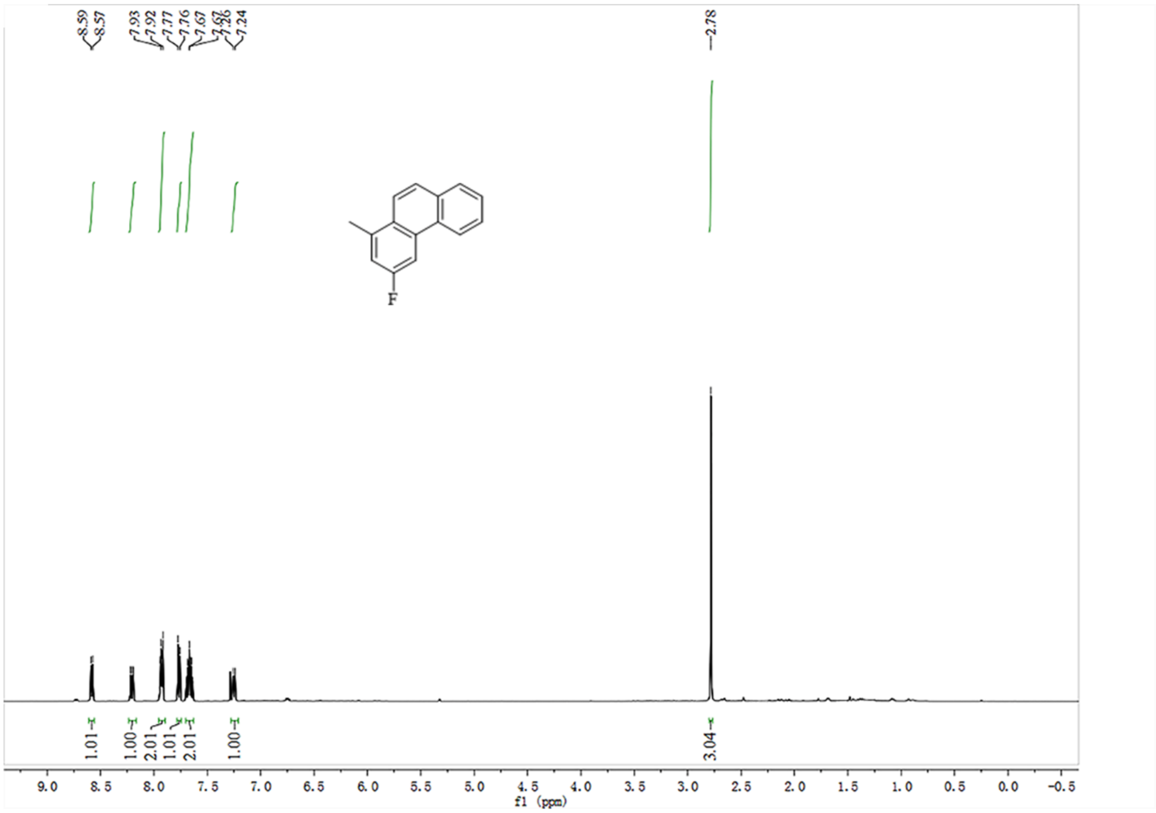
8.59
8.57
7.93
7.92
7.77
7.76
7.67
7.67
7.26
7.24
2.78
F
1.01
1.00
2.01
1.01
2.01
1.00
3.04
9.0 8.5 8.0 7.5 7.0 6.5 6.0 5.5 5.0 4.5 4.0 3.5 3.0 2.5 2.0 1.5 1.0 0.5 0.0 -0.5
f1 (ppm)

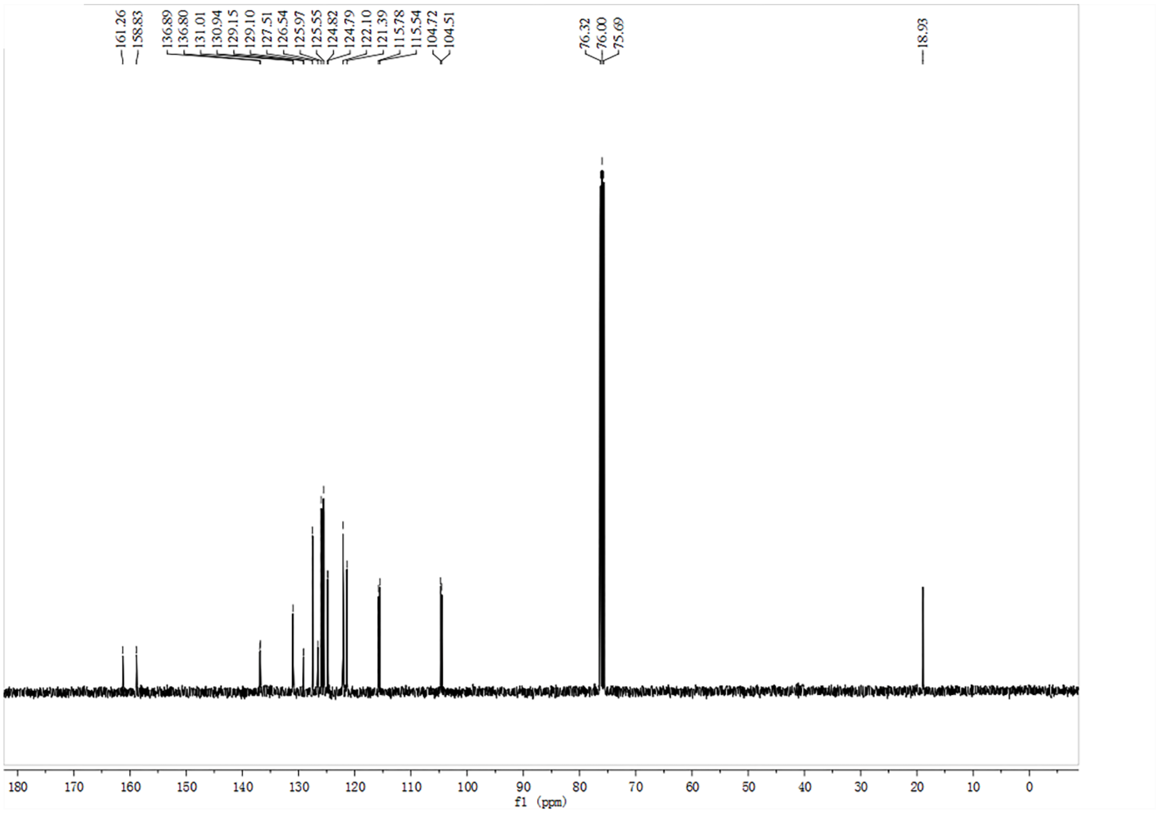
161.26
158.83
136.89
136.80
131.01
130.94
129.15
129.10
127.51
126.54
125.97
125.55
124.82
124.79
122.10
121.39
115.78
115.54
104.72
104.51
76.32
76.00
75.69
18.93
180 170 160 150 140 130 120 110 100 90 80 70 60 50 40 30 20 10 0
f1 (ppm)

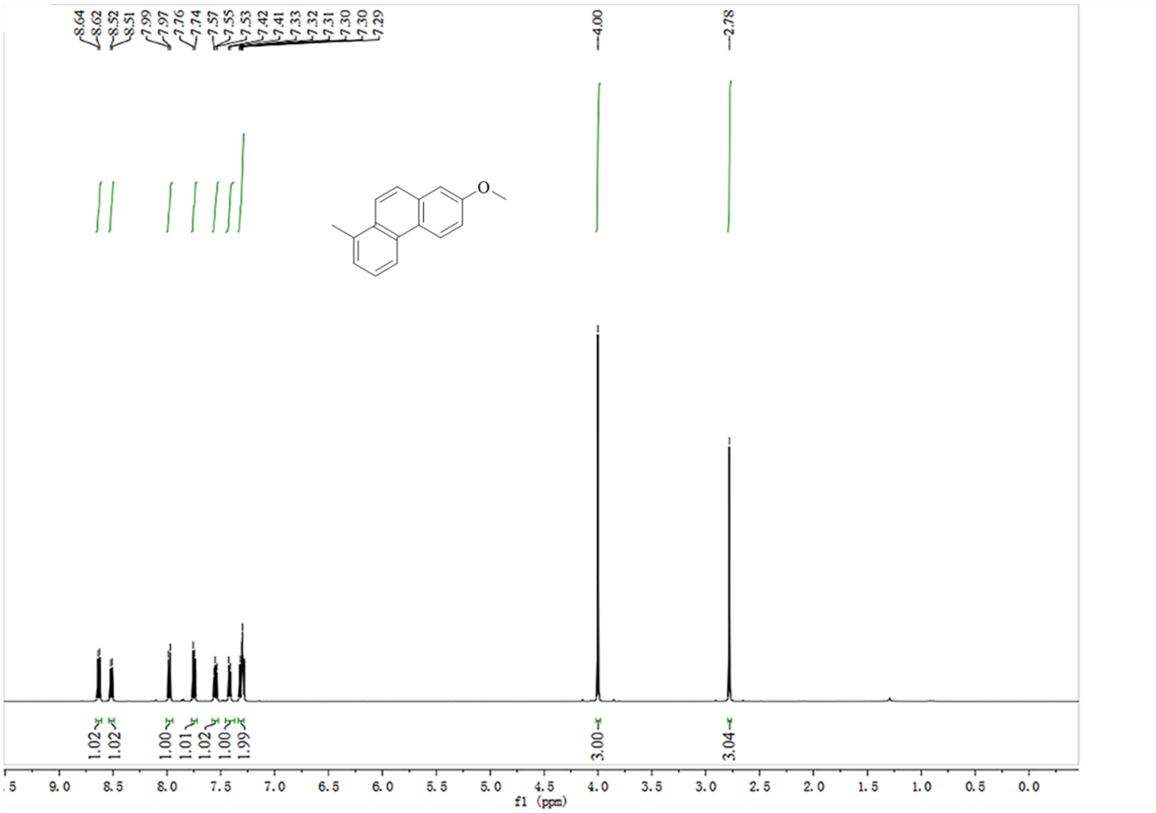
8.64
8.62
8.52
8.51
7.99
7.97
7.76
7.74
7.57
7.55
7.53
7.42
7.41
7.33
7.32
7.31
7.30
7.30
7.29
4.00
2.78
O
1.02
1.02
1.00
1.01
1.02
1.00
1.99
3.00
3.04
f1 (ppm)

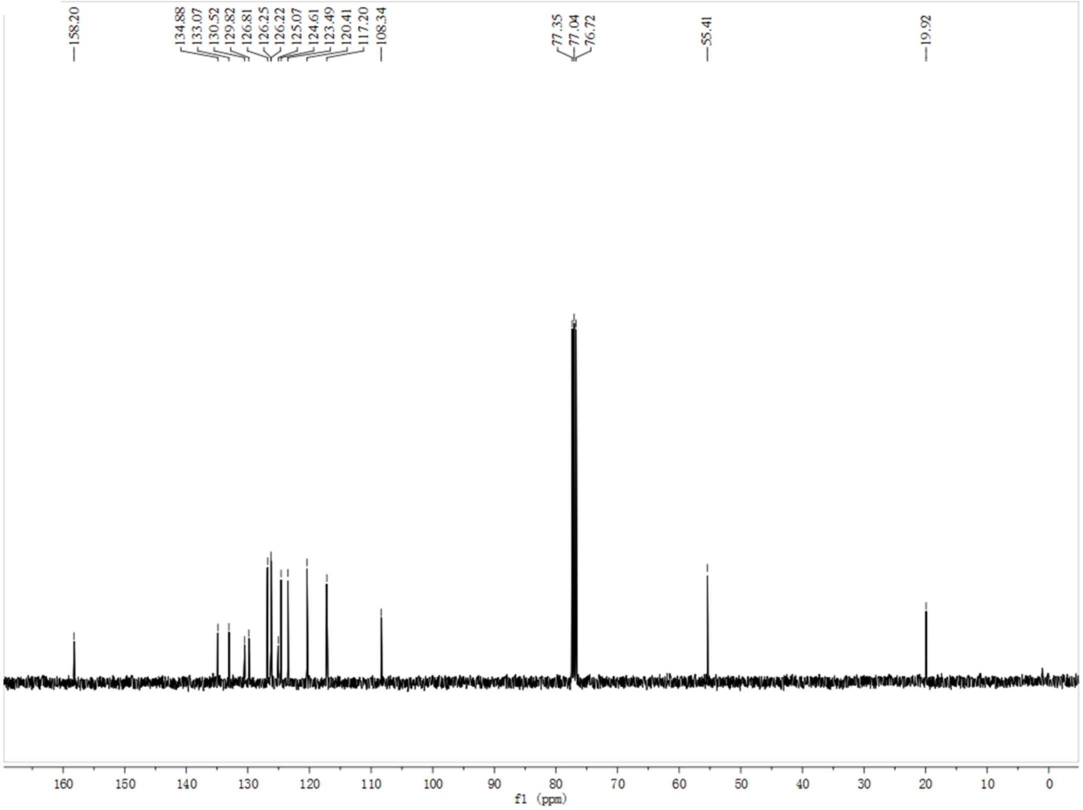
158.20
134.88
133.07
130.52
129.82
126.81
126.25
126.22
125.07
124.61
123.49
120.41
117.20
108.34
77.35
77.04
76.72
55.41
19.92
f1 (ppm)

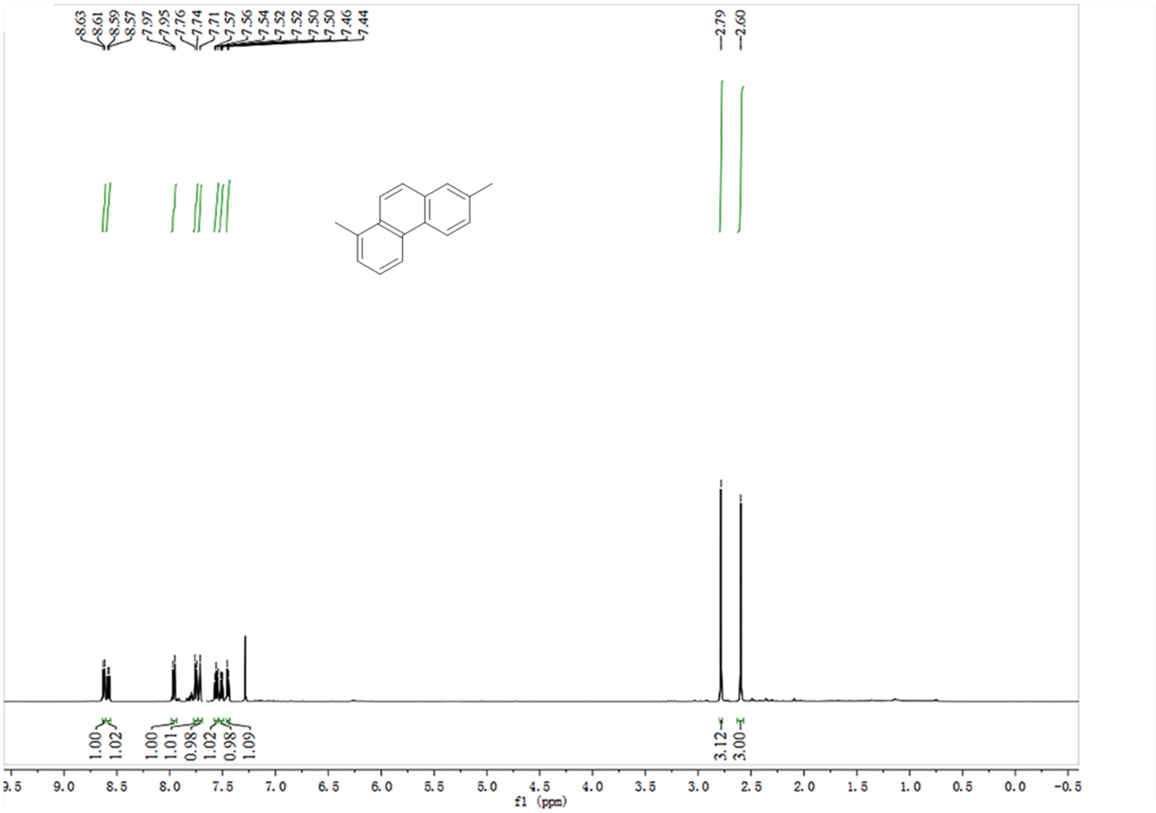
8.63
8.61
8.59
8.57
7.97
7.95
7.76
7.74
7.71
7.57
7.56
7.54
7.52
7.52
7.50
7.50
7.46
7.44
2.79
2.60
1.00
1.02
1.00
1.01
0.98
1.02
0.98
1.09
3.12
3.00
f1 (ppm)

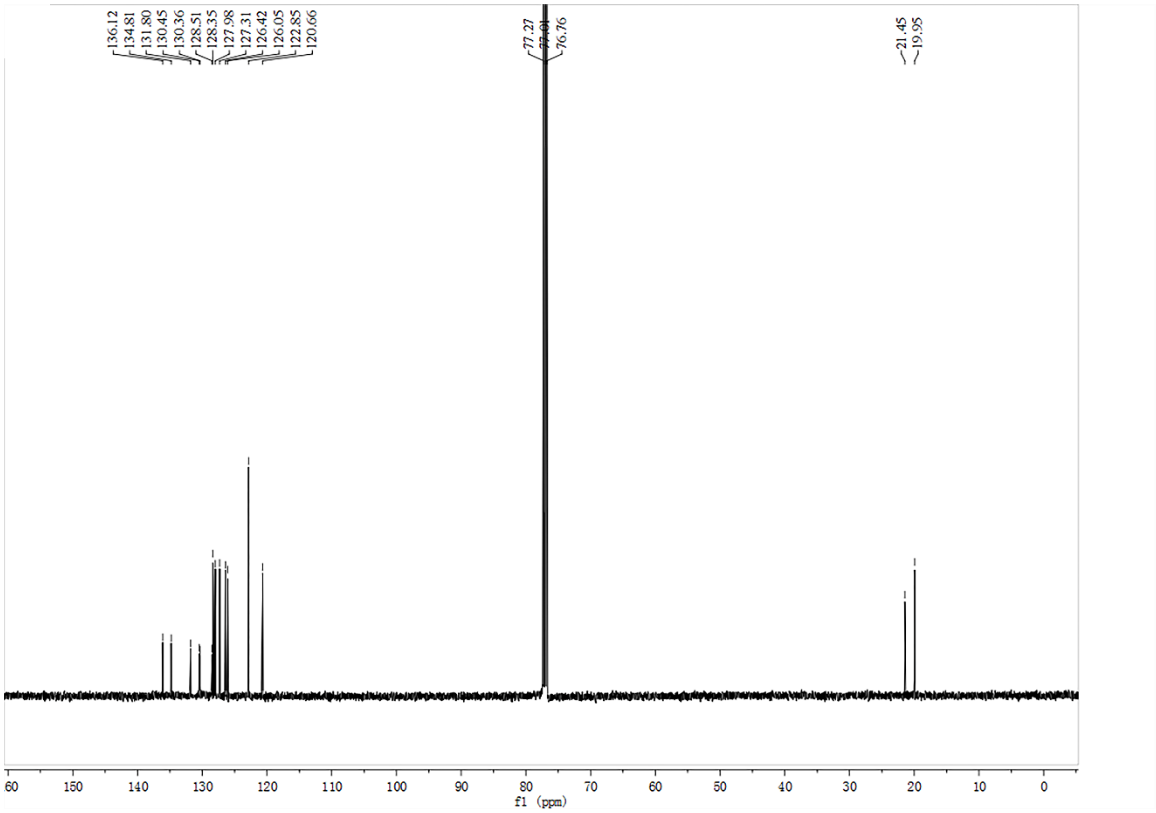
136.12
134.81
131.80
130.45
130.36
128.51
128.35
127.98
127.31
126.42
126.05
122.85
120.66
77.27
77.01
76.76
21.45
19.95
f1 (ppm)

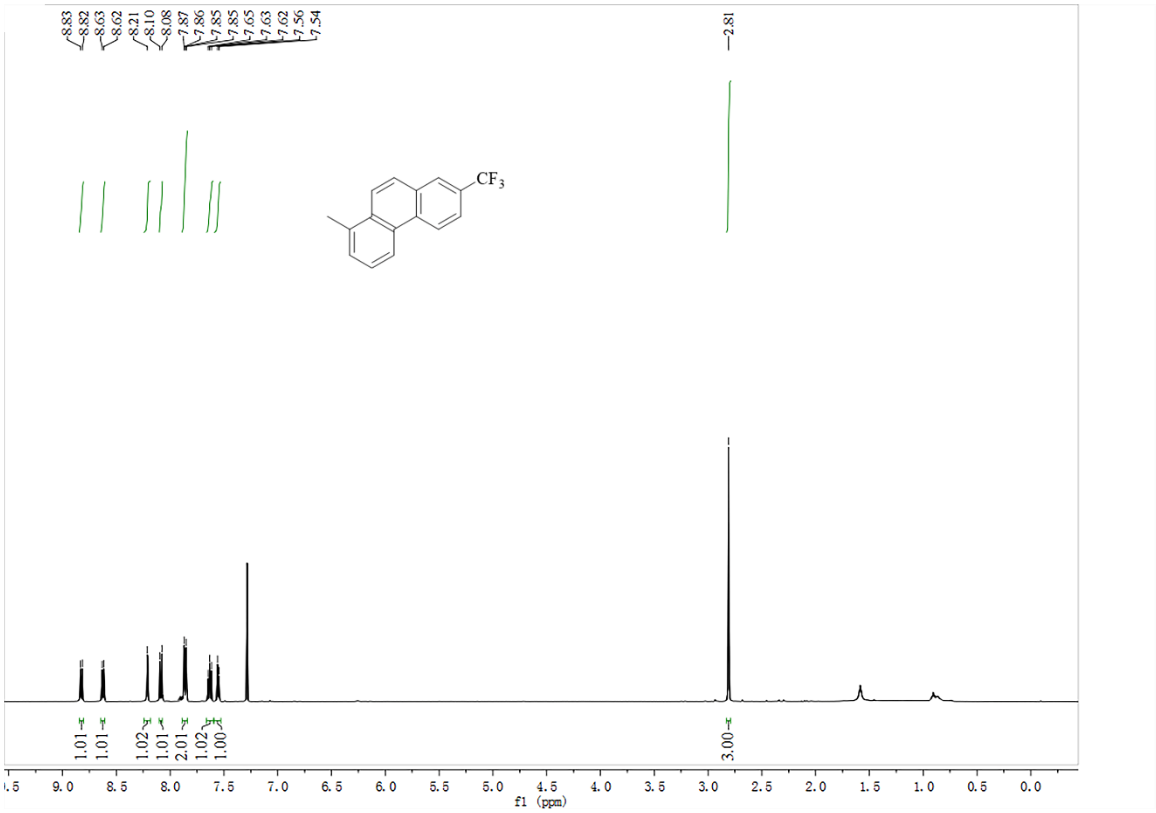

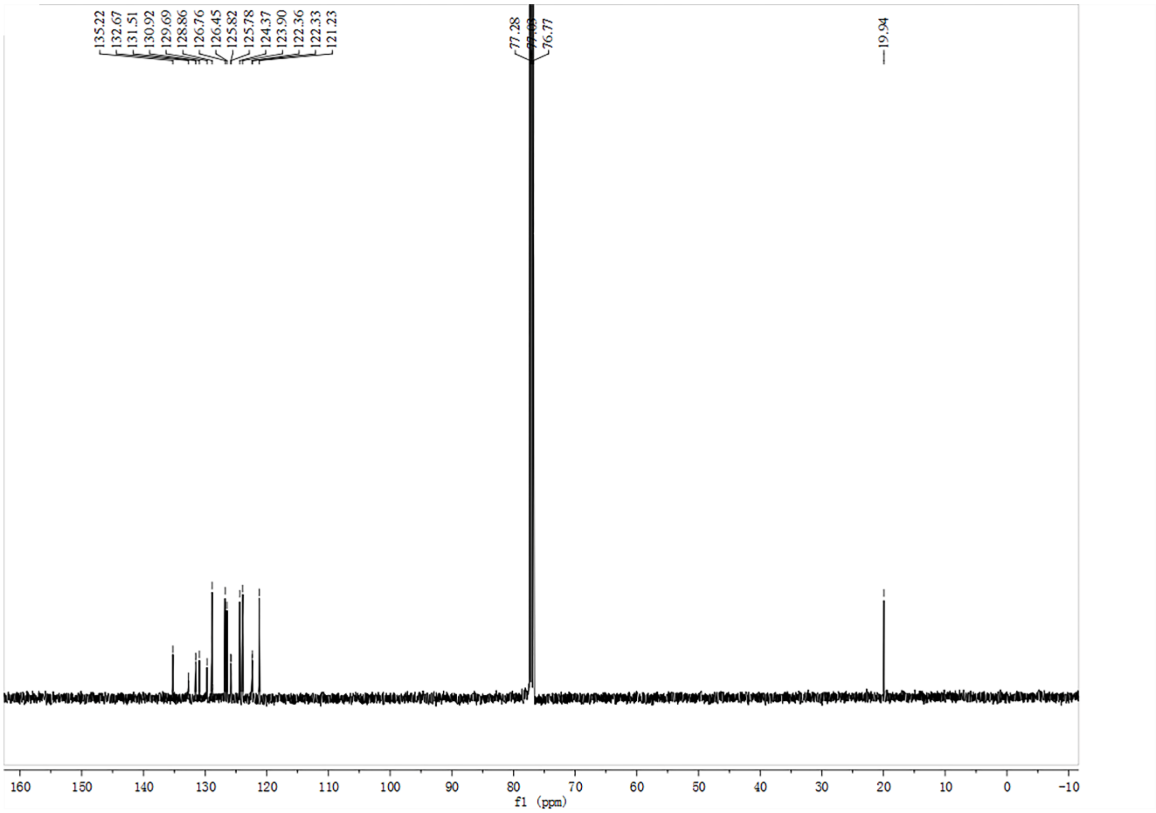

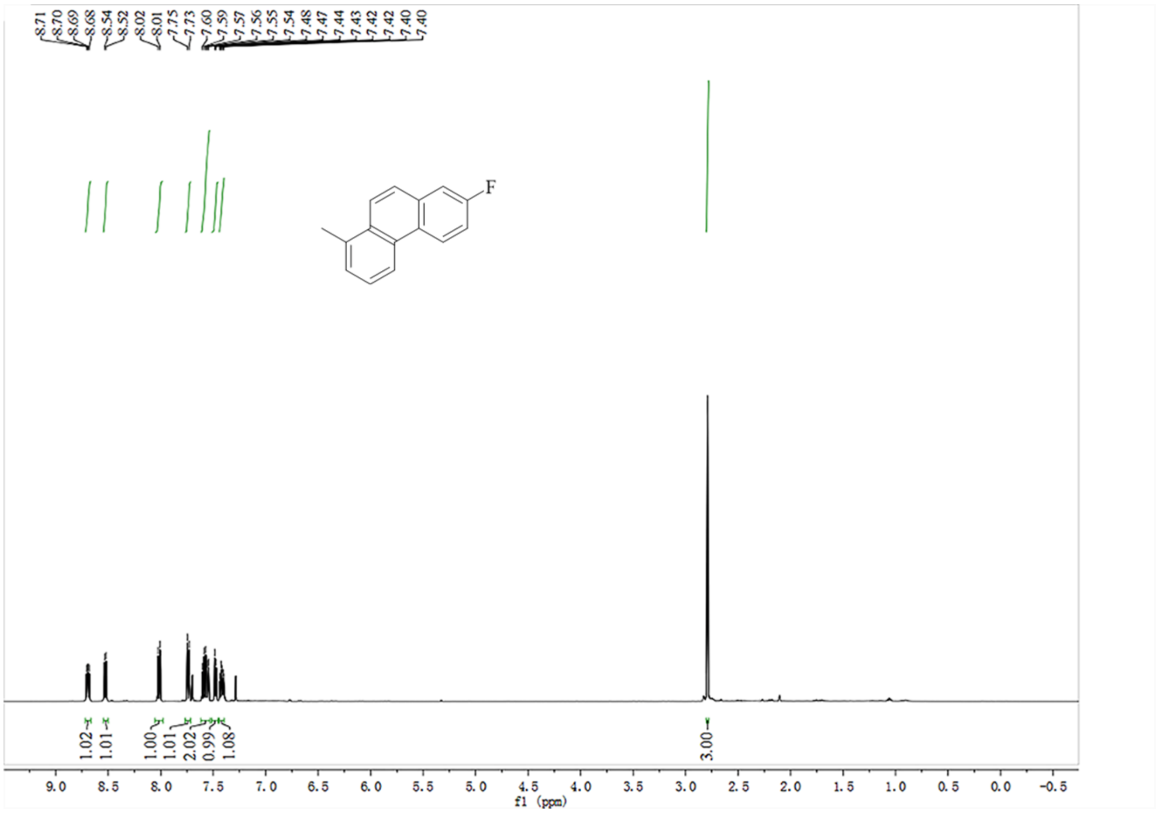
8.71
8.70
8.69
8.68
8.54
8.52
8.02
8.01
7.75
7.73
7.60
7.59
7.57
7.56
7.55
7.54
7.48
7.47
7.44
7.43
7.42
7.42
7.40
7.40
F
1.02
1.01
1.00
1.01
2.02
0.99
1.08
3.00
9.0 8.5 8.0 7.5 7.0 6.5 6.0 5.5 5.0 4.5 4.0 3.5 3.0 2.5 2.0 1.5 1.0 0.5 0.0 -0.5
f1 (ppm)

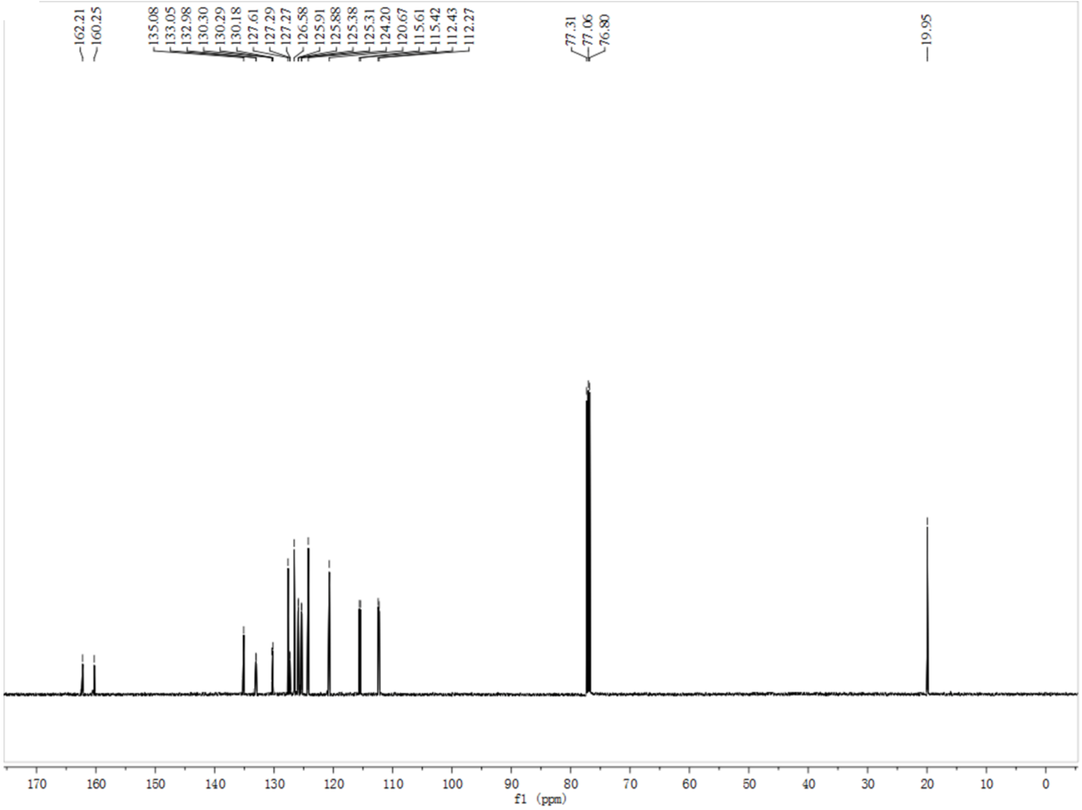
162.21
160.25
135.08
133.05
132.98
130.30
130.29
130.18
127.61
127.29
127.27
126.58
125.91
125.88
125.38
125.31
124.20
120.67
115.61
115.42
112.43
112.27
77.31
77.06
76.80
19.95
170 160 150 140 130 120 110 100 90 80 70 60 50 40 30 20 10 0
f1 (ppm)

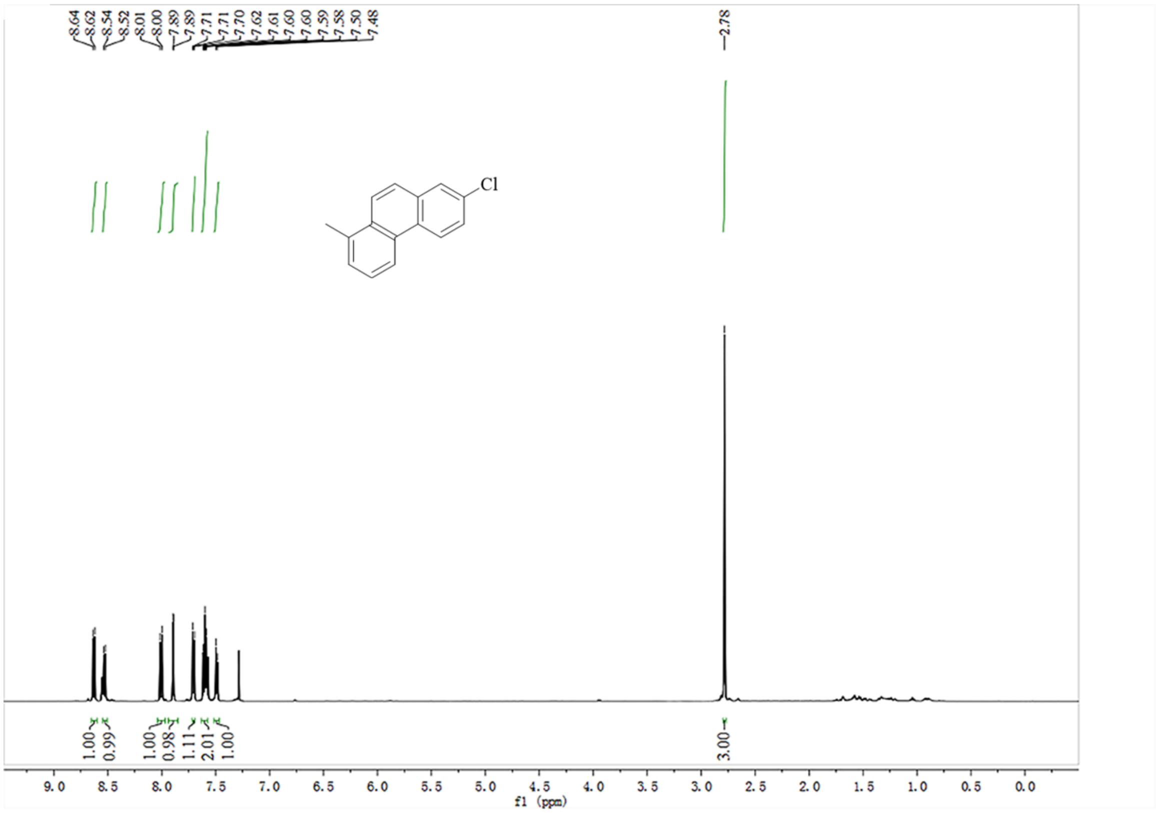

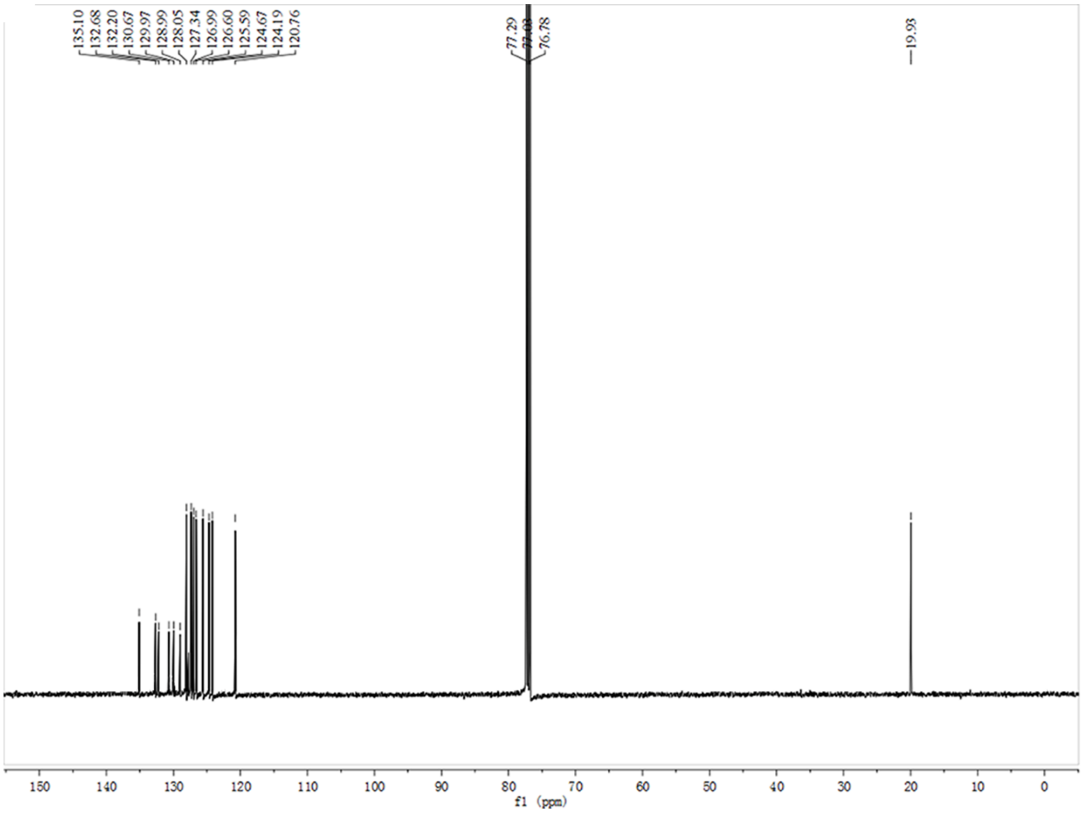

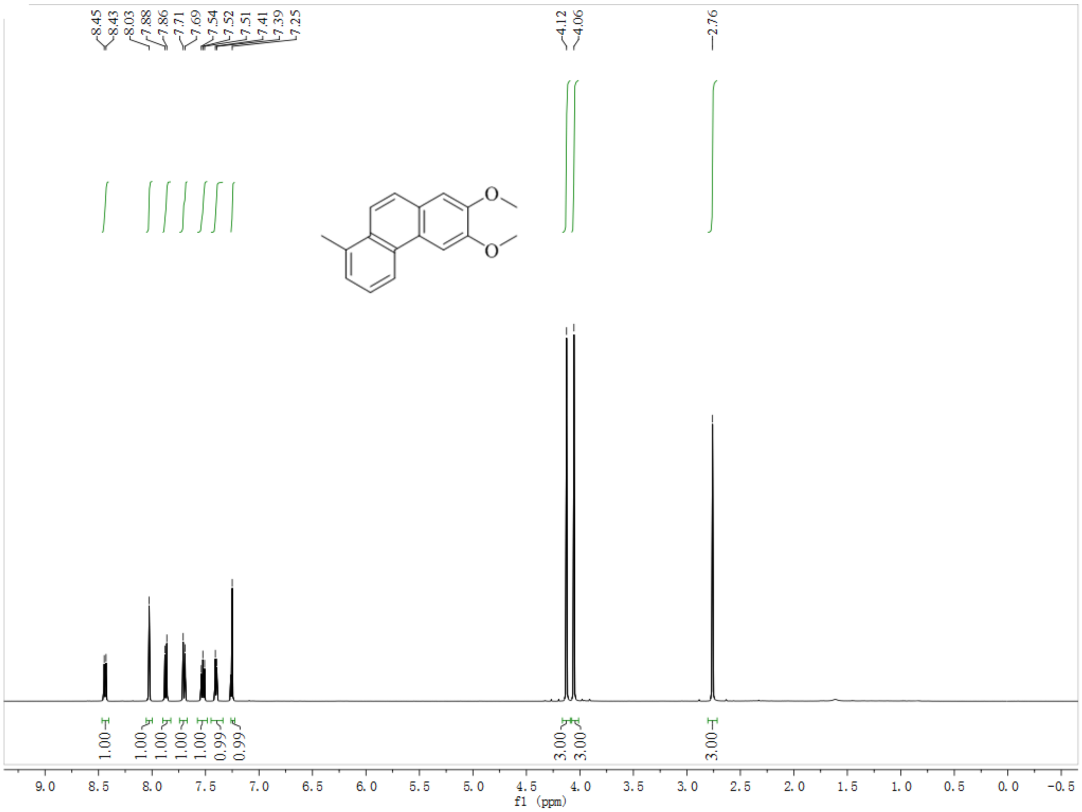
8.45
8.43
8.03
7.88
7.86
7.71
7.69
7.54
7.52
7.51
7.41
7.39
7.25
4.12
4.06
2.76
1.00
1.00
1.00
1.00
1.00
0.99
0.99
3.00
3.00
3.00
f1 (ppm)

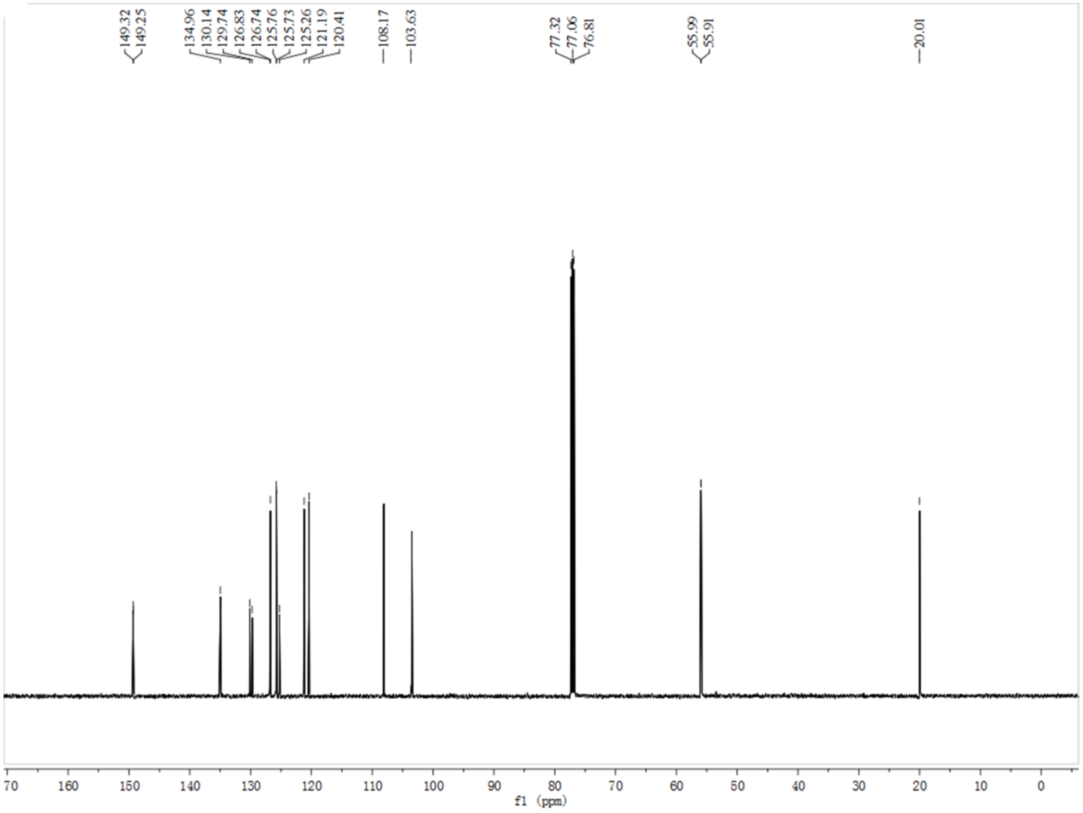
149.32
149.25
134.96
130.14
129.74
126.83
126.74
125.76
125.73
125.26
121.19
120.41
108.17
103.63
77.32
77.06
76.81
55.99
55.91
20.01
f1 (ppm)
